# Coupled intra- and interdomain dynamics support domain cross-talk in Pin1

**DOI:** 10.1074/jbc.RA120.015849

**Published:** 2021-01-13

**Authors:** Meiling Zhang, Thomas E. Frederick, Jamie VanPelt, David A. Case, Jeffrey W. Peng

**Affiliations:** 1 Department of Chemistry and Biochemistry, University of Notre Dame, Notre Dame, Indiana, USA; 2 Department of Chemistry and Chemical Biology, Rutgers University, Piscataway, New Jersey, USA

**Keywords:** allosteric regulation, multidomain protein, protein dynamic, protein-protein interaction, MD, Pin1, post-translational modification, nuclear magnetic resonance (NMR), post-translational modification (PTM), paramagnetic relaxation enhancement

## Abstract

The functional mechanisms of multidomain proteins often exploit interdomain interactions, or “cross-talk.” An example is human Pin1, an essential mitotic regulator consisting of a Trp–Trp (WW) domain flexibly tethered to a peptidyl-prolyl isomerase (PPIase) domain, resulting in interdomain interactions important for Pin1 function. Substrate binding to the WW domain alters its transient contacts with the PPIase domain via means that are only partially understood. Accordingly, we have investigated Pin1 interdomain interactions using NMR paramagnetic relaxation enhancement (PRE) and molecular dynamics (MD) simulations. The PREs show that apo-Pin1 samples interdomain contacts beyond the range suggested by previous structural studies. They further show that substrate binding to the WW domain simultaneously alters interdomain separation and the internal conformation of the WW domain. A 4.5-μs all-atom MD simulation of apo-Pin1 suggests that the fluctuations of interdomain distances are correlated with fluctuations of WW domain interresidue contacts involved in substrate binding. Thus, the interdomain/WW domain conformations sampled by apo-Pin1 may already include a range of conformations appropriate for binding Pin1's numerous substrates. The proposed coupling between intra-/interdomain conformational fluctuations is a consequence of the dynamic modular architecture of Pin1. Such modular architecture is common among cell-cycle proteins; thus, the WW–PPIase domain cross-talk mechanisms of Pin1 may be relevant for their mechanisms as well.

Modular multidomain proteins are common cell-cycle regulators in eukaryotes (1, 2). Their mechanisms often depend on transient interactions between domains serving complementary functions. Investigating these domain interactions is a necessary step toward understanding the physical basis of their functions.

This article investigates the domain interactions in human Pin1 (3), a two-domain peptidyl-prolyl isomerase (PPIase). Pin1 activity is specific for phosphorylated Ser/Thr-Pro (pS/T-P) motifs of numerous protein substrates, accelerating the *cis*-*trans* isomerization of the prolyl imide bond. Pin1 substrates include mitotic regulators, such as c-Myc (4), p53 (5), Dapk1 (6), and Cdc25C phosphatase (7), as well as neuronal proteins important for Alzheimer's disease, such as Tau (8) and APP (9).

Pin1 consists of an N-terminal WW domain (residues 1–39) that is linked by a flexible tether to a larger C-terminal PPIase domain (residues 53–163) (Fig. 1). Both domains have sites for specific pS/T-P recognition. The WW domain site consists of Loop 1 residues (Ser^16^–Arg^21^) and the side chain of Trp^34^, one of the two conserved tryptophans (Trp^11^ being the other) referred to by the WW moniker. The PPIase domain site for pS/T-P binding includes basic residues within the catalytic surface loop (residues 64–80) that arches over the hydrophobic active-site pocket.

**Figure 1:**
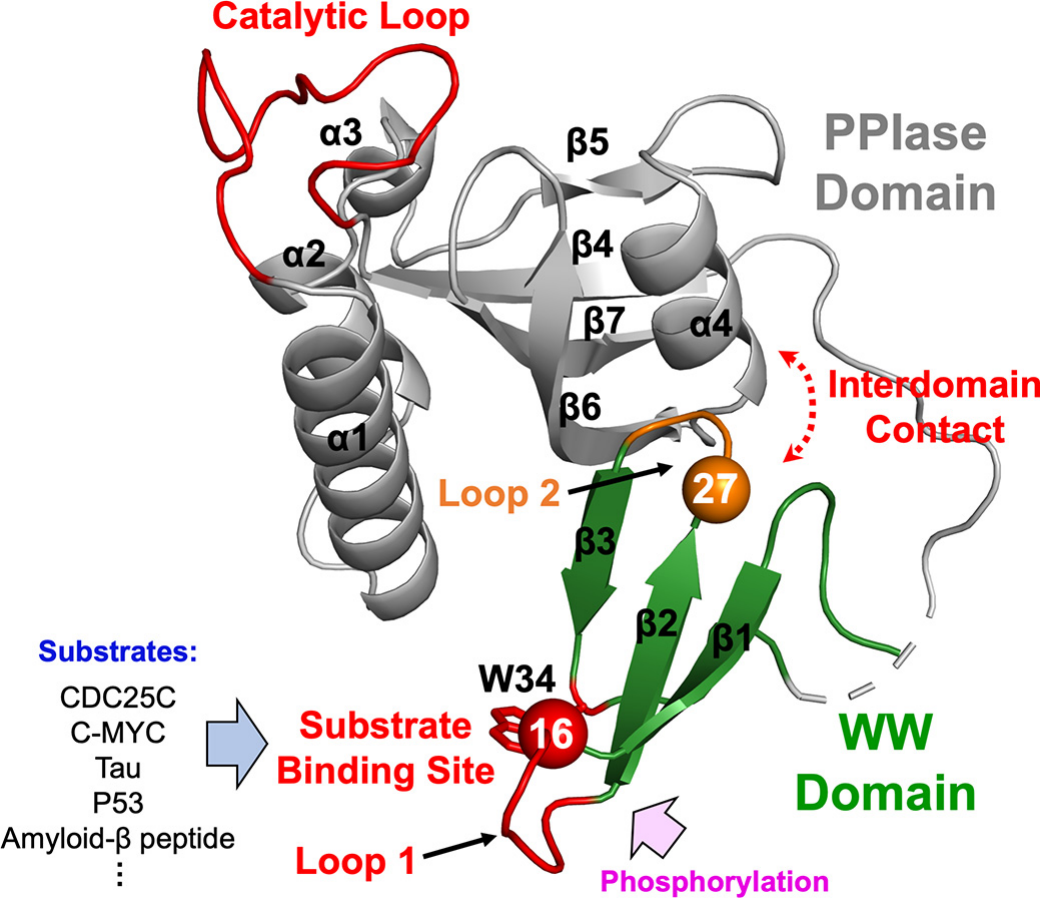
**Structural features of human Pin1 (PDB entry**1PIN**).** The N-terminal WW (*green*) and C-terminal PPIase (*gray*) domains are shown with secondary structure elements labeled. *Orange shading* denotes the WW domain Loop 2 (residues 27–29) at the interdomain interface. Residue 27 (*orange sphere*) is the MTSL (nitroxide spin label) attachment site. *Red shading* highlights the PPIase domain catalytic loop (residues 64–80) and the WW domain substrate-binding site, Loop 1, and Trp^34^. Ser^16^ (*red sphere*) is a post-translational phosphorylation site.

Previous studies of Pin1 have documented changes in PPIase activity caused by remote perturbations in the WW domain that include substitution mutations (10, 11, 12, 13) and post-translational modifications (10, 14). These long-range effects indicate the presence of a mechanism for interdomain cross-talk that remains the subject of active investigation.

A basis for such cross-talk appeared in the first Pin1 crystal structure, 1PIN (15) (Fig. 1) That structure revealed interdomain contacts (close residue proximity) formed by PPIase α4/β6 residues 137–142 on one side and WW domain Loop 2 residues 27–29 on the other. The WW domain pS/T-P site is unoccupied, whereas the PPIase active-site pocket is occupied by Ala-*cis*-Pro. The interdomain contact is partially stabilized by an interstitial PEG400 molecule. In solution, NMR studies have shown similar interdomain contacts within apo-Pin1, but they are highly transient. The transience is a consequence of the extensive relative motion between the two domains afforded by the flexible intervening linker (residues 40–52) (16, 17, 18).

Our own NMR work on Pin1 has revealed a connection between interdomain contact and interdomain cross-talk, largely via studies of Pin1 interactions with an established peptide substrate, EQPLpTPVDT, derived from the Pin1 substrate Cdc25C phosphatase from *Xenopus laevis*. Specifically, binding of the peptide substrate (pCdc25C henceforth) to Loop 1 of the WW domain (*K_D_* = 9 μm at 295 K) decreased the transient interdomain contacts between WW domain Loop 2 and PPIase domain α4/β6 residues highlighted by the 1PIN crystal structure (*e.g.* Ala^137^, Ser^138^, Phe^139^, Ala^140^, and Ser^147^) (16, 17, 19). This decrease coincided with a modest increase of *cis*-*trans* isomerase activity in the PPIase domain, as well as reduced side-chain flexibility along a conduit of conserved hydrophobic residues connecting the PPIase interdomain interface domain (α4/β6 residues in 1PIN) to the active-site pocket. These dynamic changes and the pCdc25C-induced ^15^N and ^13^C chemical shift perturbations agreed well with those of a substitution mutant that caused decreased apo-state interdomain contact while leaving substrate binding intact (I28A) (11, 13). These findings spurred our hypothesis of interdomain cross-talk as a result of allosteric communication triggered by substrate binding to the WW domain.

Fleshing out this hypothesis requires a more detailed description of the weakening of interdomain contact. However, gathering the appropriate data has proven to be nontrivial. The main challenge has been the extensive domain mobility. Such mobility has obscured the detection of ^1^H-^1^H interdomain NOEs, which could in principle map the pairwise contacts defining the interdomain interface. Consequently, our indicators of interdomain contact have been parameters such as chemical shift perturbations and spin relaxation parameters. As valuable as these parameters are, they do not directly address an obvious aspect of domain contact—interdomain separation. Consequently, we have incomplete knowledge of the residues mediating interdomain contact and how those contacts are perturbed by pCdc25C substrate binding on the opposite side of the WW domain.

We have therefore pursued new investigations of interdomain contact in Pin1 using NMR experiments to measure paramagnetic relaxation enhancements (PREs) and present our findings herein. PRE experiments involve attaching a paramagnetic nitroxide spin label to a specific Pin1 residue. Protein protons proximal to the spin label experience enhanced transverse relaxation rates (line broadening) from dipole-dipole interactions with the unpaired electron of the spin label. These interactions vary with the average of the inverse sixth power of the distance between the proton and spin label (20, 21). Thus, PREs give information similar to ^1^H-^1^H NOEs by revealing through-space contacts. The key difference is that PREs are based on the intrinsically stronger proton-electron dipolar couplings and can therefore probe longer distances (∼24 Å) (22) than ^1^H-^1^H NOEs (∼5 Å). As such, PREs are appealing for studying long-range and transient close encounters (23, 24) such as those involved in transient domain contacts (25, 26).

Here, we measured PREs due to a nitroxide spin label at the His^27^ position in Loop 2 of the WW domain. The aims of our measurements were to map the interdomain contacts sampled by apo-Pin1 and then characterize their response to pCdc25C binding. We note that our approach is distinct from the earlier PRE study of Matena *et al.* (26) that put a spin label at the Ser^18^ position in Loop 1 of the WW domain. By contrast, our spin label is at the other end of the WW domain at Loop 2 (His^27^–Thr^29^), thus allowing for substrate binding to Loop 1 while probing for possible changes in interdomain contact between Loop 2 and the PPIase domain.

In the sections below, we first describe the PREs measured for apo-Pin1, and then in the presence of saturating amounts of pCdc25C. Briefly, the PREs show a broader interdomain contact area in apo-Pin1 than previously thought. The PREs also gave direct experimental evidence for increased interdomain separation instigated by pCdc25C binding, accompanied by conformational reorganization within the WW domain. We also describe insights from an all-atom 4.5-μs molecular dynamics (MD) simulation of apo-Pin1. The 4.5-μs MD trajectory suggests that the apo-state of Pin1 already involves correlated inter- and intradomain dynamics supporting substrate-induced interdomain cross-talk. Such dynamics open the possibility that the binding of pCdc25C to the WW domain selects a subset of conformers interrelated by correlated changes of inter- and intradomain conformation and leads to the interdomain allosteric response to pCdc25C binding that we observed previously (13).

## Results

### Generation of nitroxide spin-labeled Pin1

We chose the His^27^ position in the WW domain as the site for attaching the paramagnetic nitroxide spin label, methanethiolsulfonate (MTSL). His^27^ is at the beginning of WW domain Loop 2, and its side chain is solvent-exposed. Our previous backbone ^15^N relaxation studies showed restricted mobility of the local backbone region of His^27^ relative to the WW domain β-sheet (27), thus making position 27 an attractive site for facile spin labeling and PRE data interpretation.

We introduced an H27C substitution to use established methods for attaching MTSL to cysteine residues (22, 28). To ensure exclusive MTSL labeling at position 27, we also replaced the two other WT cysteines via the substitutions C57S and C113D. The final construct was a triple mutant with a single cysteine at position 27, namely H27C/C57S/C113D-Pin1 (henceforth 3m-Pin1). From 3m-Pin1, we made two labeled samples for our PRE studies: (i) 3m-Pin1 with paramagnetic MTSL at position 27 (PARA sample) and (ii) 3m-Pin1 with the diamagnetic acetyl-MTSL at position 27 (DIA sample).

### 3m-Pin1 retains WT fold

**Figure 2:**
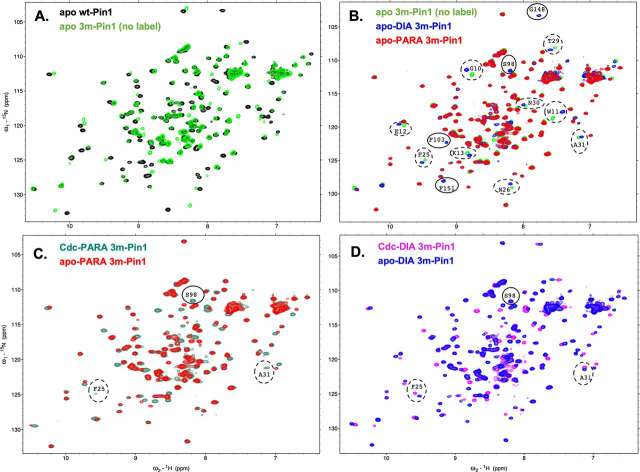
**Paramagnetic MTSL line broadening in 3m-Pin1.** Overlays of ^1^H-^15^N HSQC spectra with sample conditions as follows. *A*, apo-WT-Pin1 (*black*) and apo-3m-Pin1 (*green*); *B*, apo-3m-Pin1 (*green*), apo-DIA 3m-Pin (*blue*), and apo-PARA 3m-Pin1 (*red*). Residue cross-peaks disappearing in the PARA sample are annotated with *dashed* or *solid ovals* indicating substantial or insubstantial CSPs, respectively, in the DIA sample. *C*, pCdc25C-bound PARA 3m-Pin1 (dark *green*) and apo-PARA 3m-Pin1 (*red*). The cross-peaks for Phe^25^, Ala^31^, and Ser^98^ reappear in the PARA sample spectrum upon pCdc25C binding. *D*, pCdc25C-bound DIA 3m-Pin1 (*magenta*) and apo-DIA 3m-Pin1 (*blue*).

2D ^15^N-^1^H HSQC spectra of 3m-Pin1 show a similar dispersion of backbone NH cross-peaks to WT Pin1, indicating the same overall fold (Fig. 2*A*). We also compared the HSQC spectra of 3m-Pin1 (no label) *versus* DIA 3m-Pin1, to investigate the effects of attaching MTSL to position 27. The main effects were ^15^N-^1^H chemical shift perturbations (CSPs) confined to the WW domain (Fig. 2*B* and Table S1). This indicated negligible perturbations to the PPIase domain residues due to MTSL attachment at the domain interface (11, 13).

### 3m-Pin1 retains WT dynamic response to substrate binding

In previous Pin1 work, we observed weakened interdomain contact upon binding of the pCdc25C substrate to Loop 1 in the WW domain. The experimental parameters revealing weakened contact were backbone amide ^15^N spin relaxation rate constants ^15^N *R*_1_ = 1/*T*_1_ and *R*_2_ = 1/*T*_2_, measured for apo- and pCdc25C-complexed WT-Pin1 (13, 19). For slowly tumbling molecules, such as proteins, the rate constant combination *R*_2_-*R*_1_/2 of a given amide ^15^N provides a measure of the local rotational mobility of the corresponding NH bond vector (see “Experimental procedures”). Comparisons of the apo- and pCdc25C-complexed *R*_2_-*R*_1_/2 values revealed greater independence of domain rotational motion in the pCdc25C-complexed state (13). In other words, pCdc25C binding to the WW domain enhanced the relative rotational mobility of the two domains, an effect indicating weakened interdomain contact.

For the present study, we first needed to ensure that 3m-Pin1 retained the same functional response as WT-Pin1. We therefore conducted the same ^15^N *R*_2_-*R*_1_/2 analysis for 3m-Pin1 as done previously for WT. This included collecting new ^15^N *R*_2_-*R*_1_/2 relaxation measurements on fresh samples of WT-Pin1. We obtained similar values for WT and 3m-Pin1 for both the apo- and pCdc25C-complexed states (Fig. S1).

**Figure 3:**
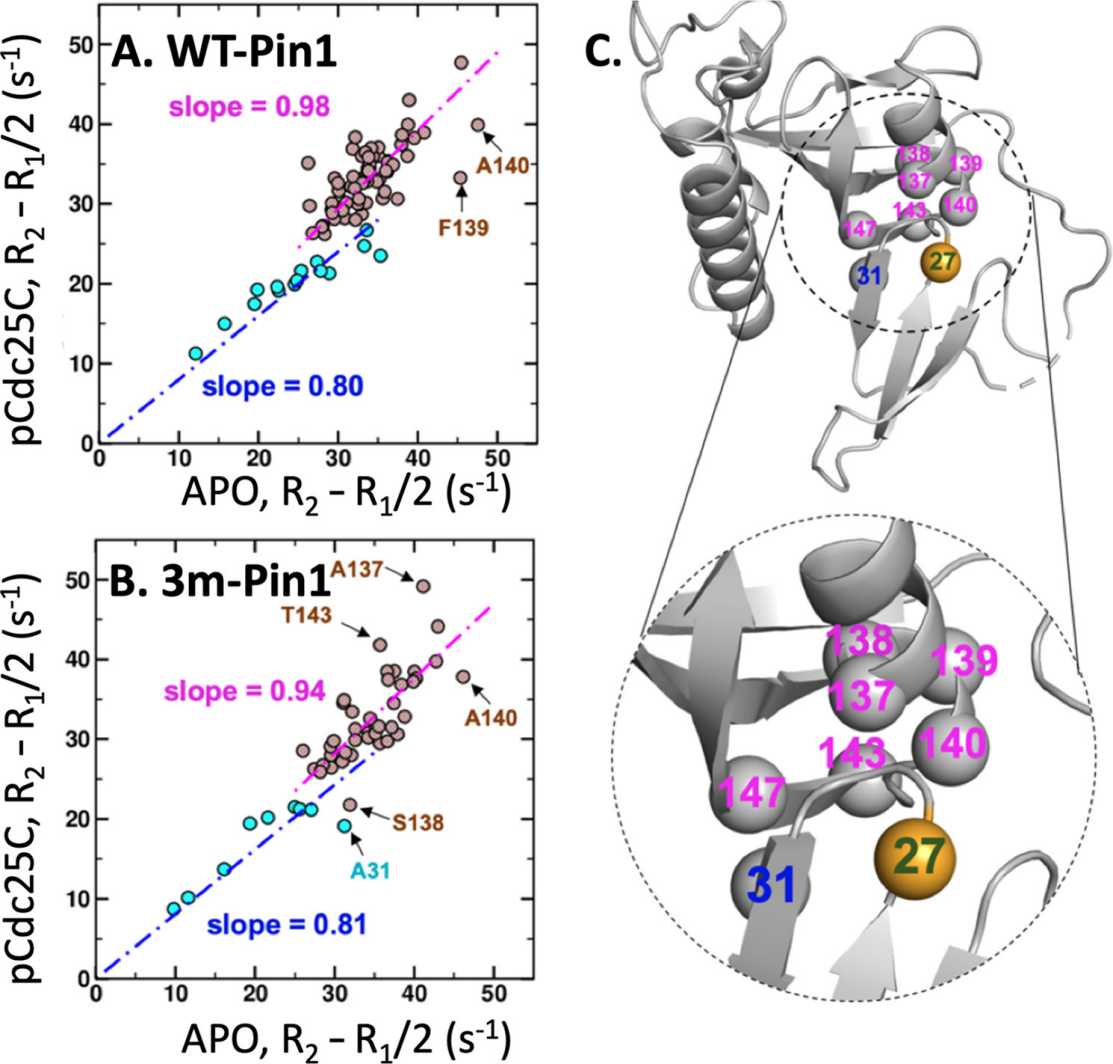
**3m-Pin1 preserves WT dynamic response to pCdc25C binding.** Linear correlation of backbone ^15^N relaxation rate constants, R_2_ − R_1_/2, for the apo-state (*horizontal*) *versus* the pCdc25C-complex state (*vertical*) for WT and 3m-Pin1. *Turquoise circles*, WW domain residues; *brown circles*, PPIase domain residues. *A*, WT-Pin1, linear regression: WW domain slope = 0.80, correlation coefficient = 0.99; PPIase domain slope = 0.98, correlation coefficient = 0.99. *B*, 3m-Pin1, linear regression: WW domain slope = 0.81, correlation coefficient = 0.98; PPIase domain slope = 0.94, correlation coefficient = 0.99. In both 3m-Pin1 and WT-Pin1, pCdc25C binding causes differential changes in domain rotational mobility, indicative of reduced interdomain contact. *C*, residues with R_2_ − R_1_/2 deviating significantly from the linear fit localize to the interdomain interface in the 1PIN crystal structure.

To address the key question of whether 3m-Pin1 retains the WT response to pCdc25C binding, we analyzed the data as described previously (13), plotting the *R*_2_-*R*_1_/2 values of the apo-state against those of the pCdc25C-complexed state, for both constructs (Fig. 3). In Fig. 3, the *R*_2_-*R*_1_/2 values from the WW and PPIase domains cluster into different regions, reflecting differences between the overall rotational mobility of the two domains. If pCdc25C binding had affected the two domains in the same way, then the dots from both domains would fall on the same line (one slope). Instead, both WT-Pin1 (Fig. 3*A*) and 3m-Pin1 (Fig. 3*B*) show *domain-specific* responses, with WW domain residues fitting to a shallower slope (∼0.80, 0.81) compared with the PPIase domain residues (∼0.94, 0.98), indicating enhanced rotational mobility of the WW domain relative to the PPIase domain and thus weaker interdomain contact upon binding of pCdc25C. Critically, Fig. 3 (*A* and *B*) shows the same domain-specific response that we observed in our previous study of WT-Pin1 (13). Therefore, 3m-Pin1 retains a defining WT response to pCdc25C binding and can therefore provide meaningful insights relevant to WT-Pin1.

Fig. 3 (*A* and *B*) also shows a handful of residues that deviate strongly from the fitted lines. In fact, these residues correspond to those we identified previously as having amplified ^15^N *R*_2_-*R*_1_/2 values indicative of exchange dynamics on the micro-millisecond time scale (11, 13, 19). For most of these residues (Ala^31^, Ser^138^, Ser^139^, and Ala^140^), binding of pCdc25C quenches the exchange dynamics, lowering the *R*_2_-*R*_1_/2 value, which causes their “dots” to fall below the fitted line. On the other hand, Ala^137^ and Thr^143^ in 3m-Pin1 show deviations above the fitted line, indicating the onset of exchange dynamics caused by pCdc25C binding, which is not apparent for WT. These residues localize to the PPIase α4/β6 interface indicated by the 1PIN crystal structure (Fig. 3*C*). We previously hypothesized that their distinctive *R*_2_-*R*_1_/2 behavior reflected exchange broadening from transient interdomain contacts (13). This hypothesis is corroborated and expanded by our new PRE results described below.

### Domain contacts in apo-Pin1 from PREs

We first investigated interdomain contact in apo-Pin1, by looking for paramagnetic broadening of NH cross-peaks going from the apo-DIA to apo-PARA 3m-Pin1 samples (Fig. 2). Some broadening was immediately apparent from visual inspection; 13 cross-peaks in the DIA spectrum “disappeared” in the PARA spectrum (Fig. 2*B* and Table S1). These disappearing cross-peaks identified residues with amide protons making close encounters with the paramagnetic MTSL spin label at WW domain position 27. Nine of these residues were in the WW domain (Gly^10^, Trp^11^, Glu^12^, Lys^13^, Phe^25^, Asn^27^, Thr^29^, Asn^30^, Ala^31^), so their disappearance reflected their co-habitation in the same domain. On the other hand, the other four residues were in the PPIase domain (Ser^98^, Phe^103^, Gly^148^, and Phe^151^). Their disappearances indicated transient contact with the WW domain.

**Figure 4:**
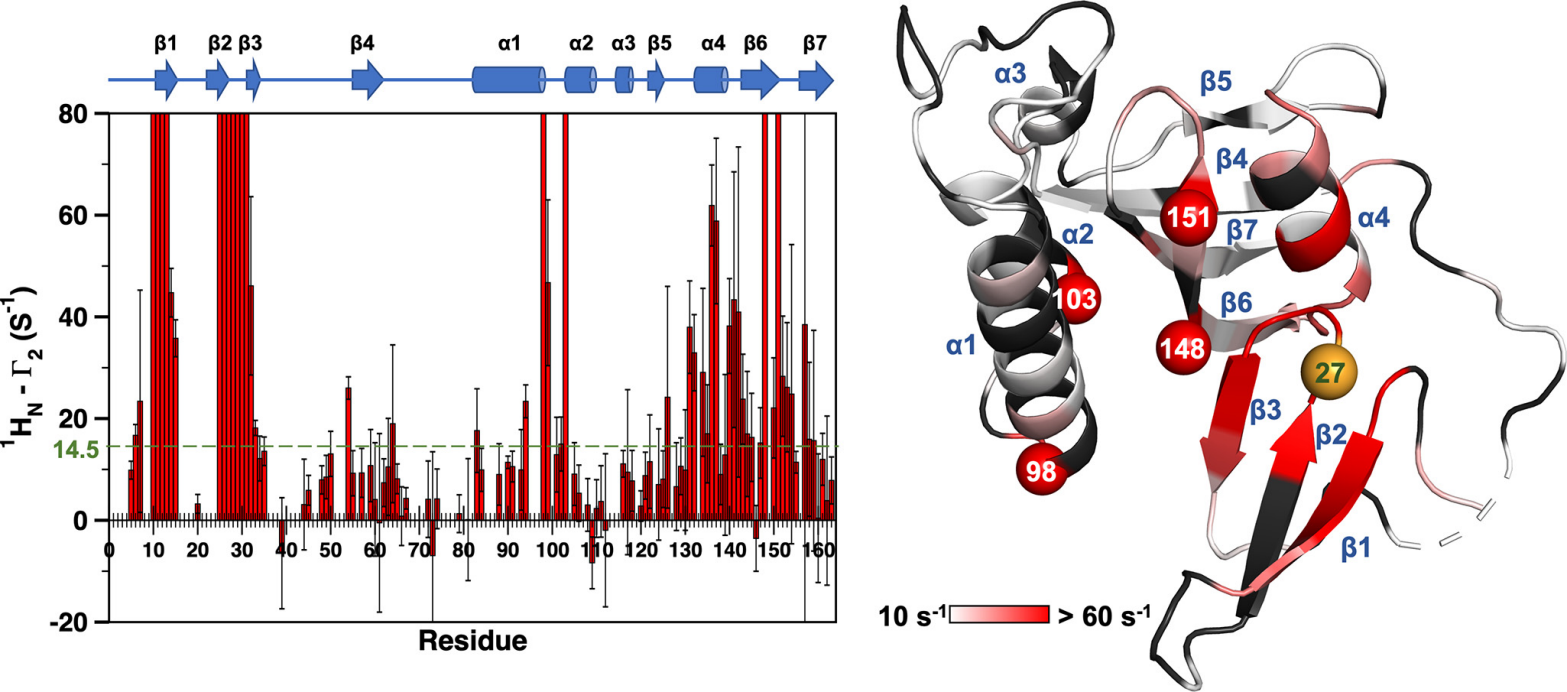
**PREs of the apo-3m-Pin1.***Left*, bar graph of the PRE rates of apo-3m-Pin1, Γ_2_ (^1^H^N^) = *R*_2, apo-PARA_(^1^H^N^) − *R*_2, apo-DIA_(^1^H^N^). Secondary structure motifs are indicated at the *top* of the bar graph. The threshold value (*dashed line*) indicates the sum of the trimmed mean and 2 times the S.D. of the filtered Γ_2_ (^1^H^N^) (14.5 rad/s) (see “Experimental procedures”). PPIase domain residues with significant Γ_2_ (^1^H^N^) values: α1 (Glu^83^, Gln^94^, Ser^98^), α1/α2 turn (Gly^99^, Asp^102^), α2 (Phe^103^), α4 (Gln^131^-Lys^132^, Phe^134^-Ala^137^, Ala^140^), α4/β6 turn (Leu^141^, Arg^142^), β6 (Thr^143^–Glu^145^, Ser^147^-Gly^148^, Val^150^–Thr^152^), β6/β7 turn (Asp^153^-Ser^154^), and β7 (His^157^-Ile^159^). *Right*, the *red gradient* denotes the amplitude of Γ_2_ (^1^H^N^) (PDB entry 1PIN) (15). *Black shading*, residues lacking Γ_2_ (^1^H^N^) values due to peak overlap or poor signal/noise ratio. The *orange sphere* is residue 27 (His^27^ in WT-Pin1), the attachment site for the nitroxide spin label MTSL and its diamagnetic counterpart (acetyl-MTSL). The *red*, *numbered spheres* are PPIase domain residues disappearing in the presence of paramagnetic MTSL (apo-PARA sample).

For a more complete analysis, we measured amide proton transverse relaxation rate constants *R*_2_ (^1^H^N^) for apo-states of PARA and DIA 3m-Pin1 (see “Experimental procedures”) (24). Sequence-specific PREs, denoted by Γ_2_(^1^H^N^), were the differences Γ_2_(^1^H^N^) = *R*_2, apo-PARA_(^1^H^N^) − *R*_2, apo-DIA_(^1^H^N^) and are shown in Fig. 4. Significant Γ_2_(^1^H^N^) values were identified as those deviating from the trimmed mean by more than 2 S.D. values. The largest Γ_2_(^1^H^N^) from curve fitting was 61.9 ± 8.0 rad/s for Asp^136^. The larger values implicit in the disappearance of the 13 cross-peaks appear as “overflow” bars in Fig. 4.

Fig. 4 reveals two regions of PPIase residues involved in transient interdomain contact. One region starts at α4 and ends in first half of β7 (designated α4/β6/β7 henceforth). This region includes two of the four disappearing PPIase residues, Gly^148^ and Phe^151^. It also includes Ala^140^ and Leu^141^ at the α4/β6 juncture, residues with ^15^N/^1^H chemical shifts that have been shown to be diagnostic of interdomain contact (13). The α4/β6/β7 contact region is also compatible with the residues showing enhanced *R*_2_-*R*_1_/2 values sensitive to pCdc25C binding (outliers in Fig. 3 (*A* and *B*)) and the 1PIN crystal structure (Fig. S2) (15), which places these α4/β6/β7 residues across from the MTSL spin label site at the His^27^ position in Loop 2 of the WW domain.

Fig. 4 shows an unexpected, second interdomain contact region, defined by the large Γ_2_(^1^H^N^) values for PPIase residues Glu^83^, Gln^94^, and Ser^98^ in α1, Gly^99^ and Asp^102^ in the α1/α2 turn, and Phe^103^ in α2. This second contact region (referred to as α1/α2 henceforth) includes Ser^98^ and Phe^103^, the other two “disappearing” PPIase residues. Significantly, as seen in the 1PIN crystal structure, the relative locations of α1/α2 sites and α4/β6/β7 sites within the PPIase domain are such that proximity to the spin label by one cohort excludes the other (Fig. S2) (15). The α1/α2 sites thus expand the range of interdomain contacts in apo-Pin1 beyond what was previously supposed (Fig. S2). The broader PPIase/Loop 2–interacting surface could derive from the intrinsic flexibility of the interdomain linker (∼10 residues). The plausibility of this hypothesis is supported by our all-atom MD simulations (see below).

We note that the amino acid content of two PPIase domain contact regions, α4/β6/β7 and α1/α2, bolsters the hypotheses of our previous study of I28A-Pin1, which suggested hydrophobic interactions mediating contact between Loop 2 and the PPIase domain (11). The four PPIase residues that disappeared in apo-PARA (Ser^98^, Phe^103^, Gly^148^, and Phe^151^) are either hydrophobic or adjacent to a hydrophobic residue, making them potential interacting partners for Ile^28^.

### Contact changes upon binding pCdc25C

Next, we investigated the effects of pCdc25C binding on the apo-state interdomain contacts, by measuring PREs for 3m-Pin1 under saturating amounts of pCdc25C substrate.

Some changes induced by pCdc25C were obvious from differences between the PARA spectra from apo- and Cdc-3m Pin1 (Fig. 2*C*) and the corresponding DIA spectra (Fig. 2*D*). Notably, three residues that had been completely broadened out in apo-PARA 3m-Pin1—Phe^25^ and Ala^31^ in the WW domain and Ser^98^ in the PPIase domain—reappeared in Cdc-PARA 3m-Pin1.

**Figure 5:**
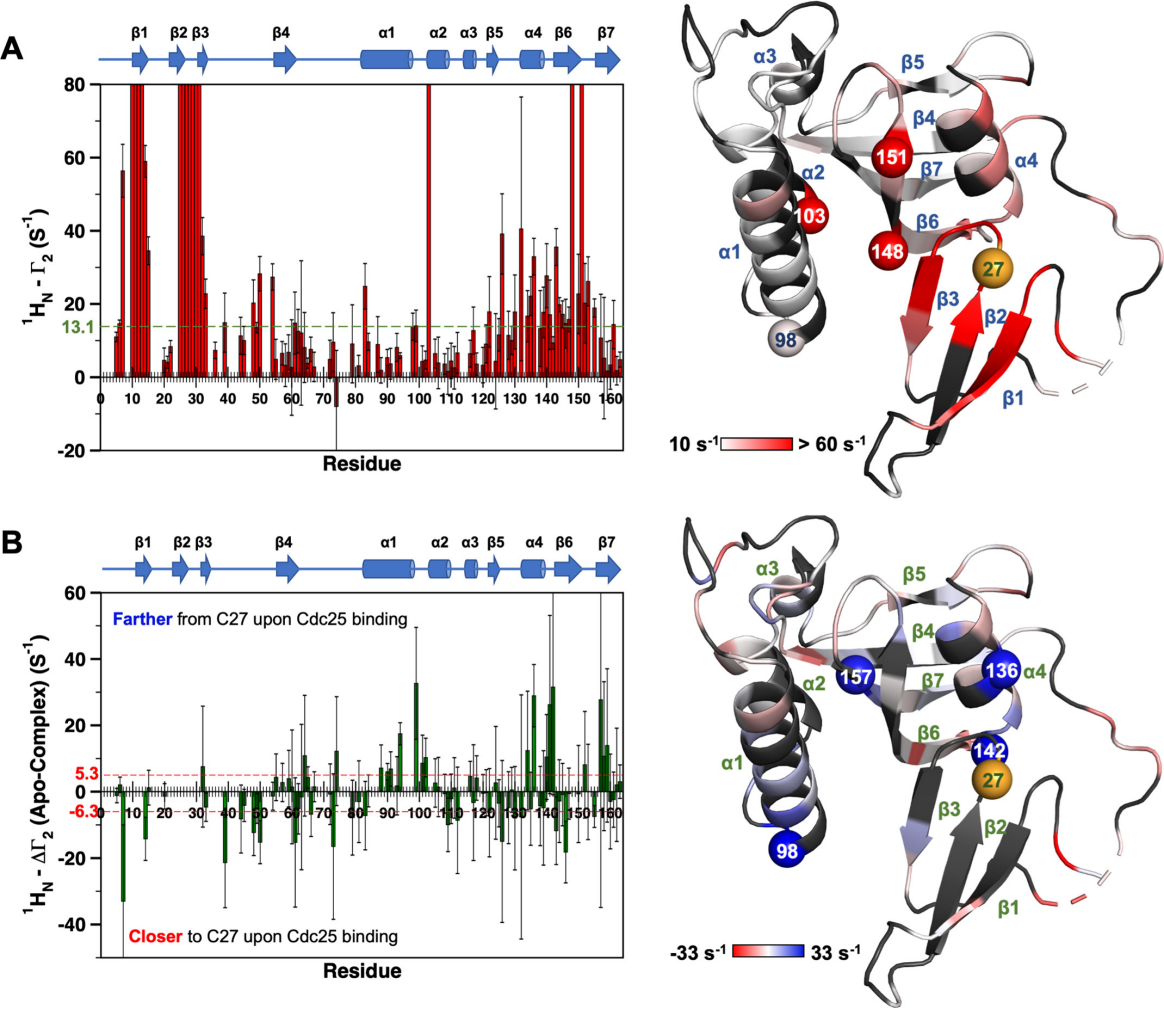
**pCdc25C binding increases interdomain separation.***A*, *top left*, PRE values, Γ_2_(^1^H^N^) = *R*_2, CDC-PARA_(^1^H^N^) − *R*_2, CDC-DIA_(^1^H^N^) *versus* sequence for pCdc25C-complexed 3m-Pin1 with secondary structure elements across the *top*. The *dashed green line* indicates the significance threshold of 2 S.D. values above the trimmed mean (13.1 rad/s). *Top right*, 1PIN structure with *red gradient shading* indicates the location and relative magnitudes of Γ_2_(^1^H^N^) (15); *red spheres* denote PPIase domain residues that disappear in apo-PARA Pin1. *B*, *bottom left*, changes in Γ_2_(^1^H^N^) caused by pCdc25C binding, ΔΓ_2_(^1^H^N^) = Γ_2,apo_(^1^H^N^) − Γ_2,CDC_(^1^H^N^). The *red dashed line* indicates the significance threshold of 2 S.D. values beyond the trimmed mean (+5.3 and −6.3 rad/s). *Bottom right*, 1PIN structure with *blue-to-red gradient shading* for ΔΓ_2_ (^1^H^N^); *blue/red*, decreased/increased Γ_2_(^1^H^N^), respectively, in the pCdc25C complexed state. *Blue spheres*, PPIase domain residues showing the largest reduction of ΔΓ_2_(^1^H^N^) upon pCdc25C binding. *Black shading*, residues lacking Γ_2_(^1^H^N^) values due to peak overlap or poor signal/noise ratio. *Orange sphere*, MSTL attachment site at position 27 in the WW domain (His^27^ in WT-Pin1).

We obtained quantitative PREs (Γ_2_(^1^H^N^) values) for the Cdc-3m-Pin1 samples, using the same *R*_2_ (^1^H^N^) experiments described for the apo-state. Fig. 5*A* shows the resulting profile of Γ_2_(^1^H^N^) *versus* sequence. The profile shape resembles that of apo-3m-Pin1, albeit with generally smaller Γ_2_(^1^H^N^) magnitudes. The changes induced by pCdc25C binding are more apparent in Fig. 5*B*, which plots the differences ΔΓ_2_ = Γ_2,APO_(^1^H^N^) – Γ_2,CDC_(^1^H^N^). The plot reveals reduced PREs (ΔΓ_2_ > 0) for residues in the two domain contact regions, α4/β6/β7 and α1/α2, in the pCdc25C-complexed form and, therefore, greater distance of these sites from the spin label at position 27. The sites showing the most prominent reductions include Ser^98^ in the α1/α2 region and Asp^136^, Arg^142^, and His^157^ in the α4/β6/β7 region, indicating pCdc25C-induced increases of the interdomain distances D_H27Cα–S98Cα_, D_H27Cα–D136Cα_, D_H27Cα–R142Cα_, and D_H27Cα–H157Cα._

Fig. 5*B* also shows significant ΔΓ_2_ within the WW domain itself. These changes include negative values (ΔΓ_2_ < 0) (*i.e.* Γ_2,APO_(^1^H^N^) < Γ_2,CDC_(^1^H^N^)), indicating substrate-induced decreases of *intradomain* distances, suggesting perturbations of WW domain conformation. We note that these intradomain conformational perturbations coincide with the increased interdomain distances described above. WW domain residues with negative ΔΓ_2_ include Leu^7^, Arg^14^, and Gly^39^. Notably, Leu^7^ and Arg^14^ are parts of two distinct, conserved hydrophobic cores (core I: Leu^7^, Trp^11^, Tyr^24^, and Pro^37^; core II: Arg^14^, Tyr^23^, and Phe^25^), and their NH chemical shifts are diagnostic of substrate binding (13). Furthermore, the changes in intradomain distances indicated by Leu^7^ and Arg^14^ are consistent with the substrate-induced compaction (increased concavity) of WW domain noted by early X-ray and NMR structural studies (29, 30).

In summary, our PREs gave the following new insights: (i) apo-Pin1 has a larger area of transient interdomain contacts, including the more canonical α4/β6/β7 region and the α1/α2 region revealed herein, and (ii) pCdc25C binding to the WW domain reduces apo-state interdomain contact, the most pronounced changes being increases of interdomain distances D_H27Cα–S98Cα_, D_H27Cα–D136Cα_, D_H27Cα–R142Cα_, and D_H27Cα–H157Cα_ and simultaneous decreases of intradomain (WW domain) distances, D_H27Cα–L7Cα_, D_H27Cα–R14Cα_, and D_H27Cα–G39Cα_.

### All-atom MD simulations of apo-Pin1

The PPIase domain PREs in apo-3m-Pin1 revealed two regions making transient contact with Loop 2 in the WW domain: α1/α2 and α4/β6/β7. The strongest responders included Ser^98^ and Phe^103^ in α1/α2 and Gly^148^ and Phe^151^ in α4/β6/β7. We wanted to explore plausible Pin1 conformations that could produce these responses. Accordingly, we performed explicit solvent MD simulations of apo-Pin1 using AMBER 16 (31).

To minimize biasing the domain contact surface (*e.g.* the closed conformation of the 1PIN crystal structure (15)), we used the first model from the NMR solution structure deposition (PDB entry 1NMV (17)) as our starting structure. This model has the two domains well-separated. The simulation temperature was 300 K, and the production run was ∼4.5 μs. Snapshots were saved every 200 ps, producing a time series of 22,400 conformations (see “Experimental procedures”).

The water model was critical for simulating interdomain motion. Specifically, we used the three-charge, four-point rigid water model (OPC) (32, 33) that was developed to improve the simulations of intrinsically disordered proteins and/or protein regions. By using OPC waters, our simulation sampled both interdomain association and separation. By contrast, our initial simulation attempts using the more standard TIP3P model led to domain association but no separation.

### Consistency between MD interdomain distances and PREs

**Figure 6:**
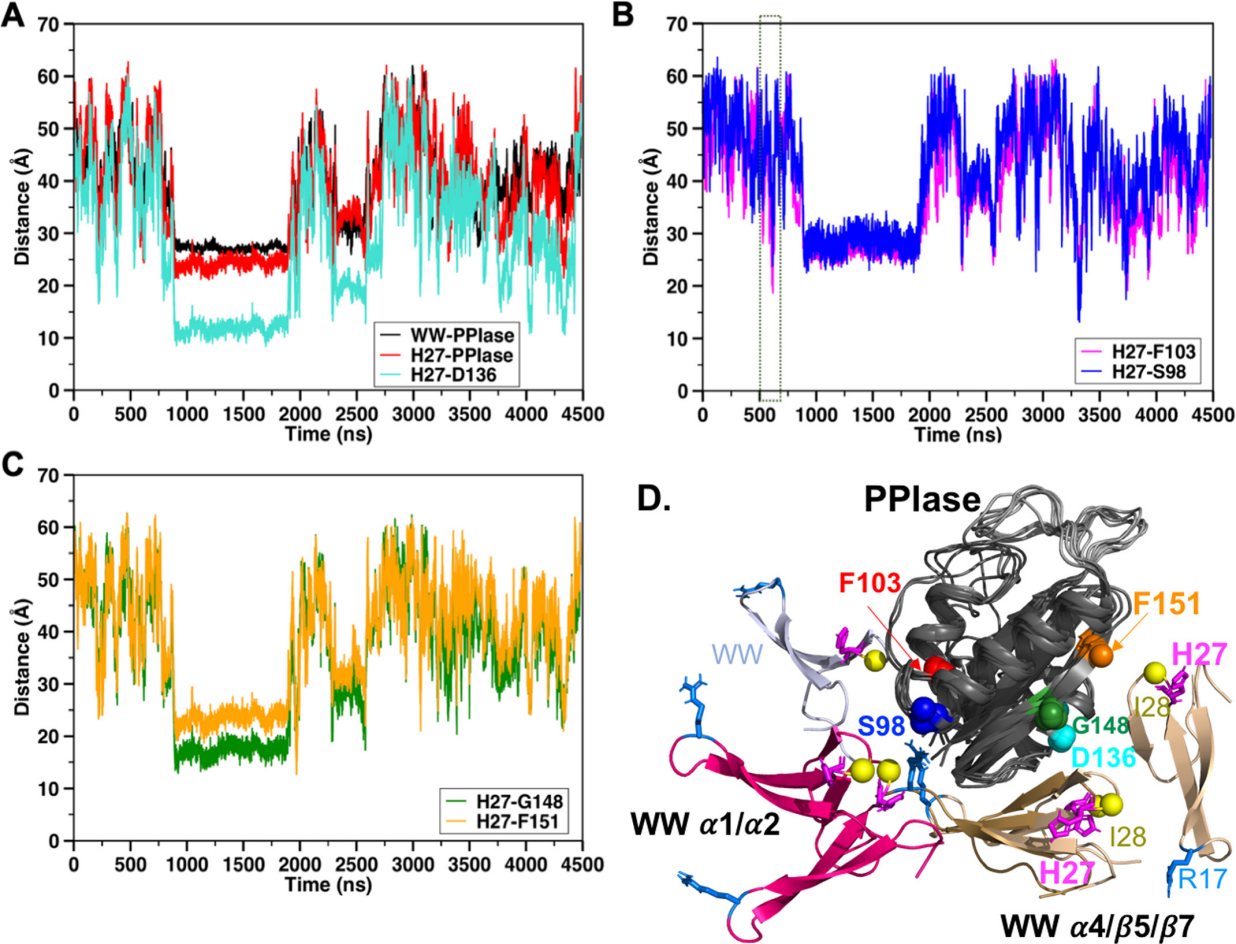
**MD simulations suggest multiple interdomain contacts.***A–C*, fluctuations of diagnostic interdomain distances throughout the 4.5-μs MD trajectory, where *WW* and *PPIase* denote the centers of mass of the respective domains. The *dashed rectangle* in *B* is enlarged in Fig. 7*A* (*bottom*). *D*, MD snapshots aligned by their PPIase domain (*dark gray*). The snapshots are configurations with H27Cα, the spin label position, at its closest distance to the Cα of other PPIase domain residues that either vanished in the apo-PARA 3m-Pin1 sample (Ser^98^, Phe^103^, Gly^148^, and Phe^151^) or had the largest measurable Γ_2_(^1^H^N^) (Asp^136^). The configurations are distinguished by WW domains *colored* as follows: Gly^148^ Cα and Phe^151^ Cα (*wheat*), Ser^98^ Cα and Phe^103^ Cα (*hot pink*); Asp^136^ Cα (sand). Also shown (in *blue white*) is the configuration at 610.6 ns with Phe^103^ closer to His^27^ Cα than Ser^98^ Cα. *Spheres* indicate the PPIase residues that showed the most prominent PREs in the apo-PARA 3m-Pin1 sample: Ser^98^ (*blue*), Phe^103^ (*red*), Asp^136^ (*turquoise*), Gly^148^ (*dark green*), and Phe^151^ (*orange*). In the WW domain, the *yellow spheres* indicate Ile^28^ in Loop 2, whereas *marine sticks* indicate Arg^17^ in Loop 1, the substrate-binding site in the WW domain.

We investigated the MD time series of interdomain distances, including the distance between (i) the domain centers of mass, (ii) H27Cα (MTSL spin label position) and the PPIase center of mass, and (iii) the Cα atoms of His^27^ and residues showing the most prominent PREs in apo-3m-Pin1, namely D_H27Cα–D136Cα_, D_H27Cα–G148Cα_, and D_H27Cα–H151Cα_ in the α4/β6/β7 region and D_H27Cα-S98Cα_ and D_H27Cα-F103Cα_ in the α1/α2 region (Fig. 6).

^1^H^N^ PREs are observable for distances up to ∼24 Å from the spin label (22, 34). Gratifyingly, the five Cα–Cα distances in Fig. 6 sampled values <24 Å over the course of the trajectory, with their minimum values having an average of ∼13 Å. The breadth and relative likelihood of distance values are shown in the histograms in Fig. S3, for D_H27Cα-D136Cα_, D_H27Cα−G148Cα_, D_H27Cα−H151α_, D_H27Cα-S98Cα_, and D_H27Cα-F103Cα_. Thus, the MD simulation samples close interdomain contacts indicated by the PREs of apo-Pin1.

**Figure 7:**
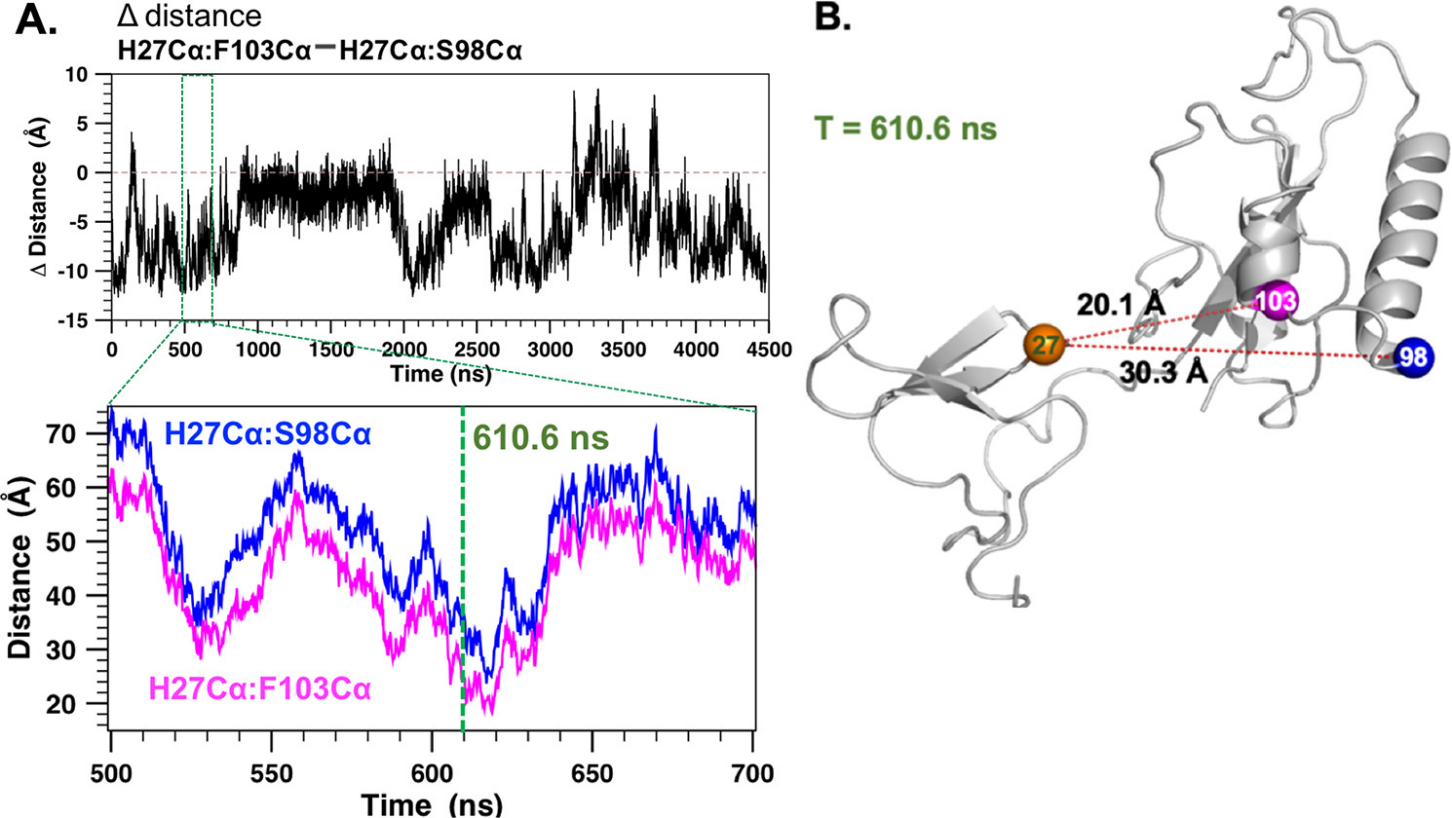
**MD of apo-WT-Pin1 captures interdomain conformations supporting PRE changes induced by pCdc25C binding.***A*, *top*, time series of distance differences (D_H27Cα-F103Cα_ – D_H27Cα-S98Cα_). A (*bottom*), *zoom-in view* of Fig. 6*B* showing a trajectory segment where interdomain distance D_H27Cα-F103Cα_ < D_H27Cα-S98Cα_, which could explain the larger PRE observed for Phe^103^ than Ser^98^ in the pCdc25C-bound Pin1. This suggests that the pCdc25C-bound conformation could preexist as a sparse population in the apo-ensemble. *B*, an MD snapshot at 610.6 ns (also indicated in *A* (*bottom*)) with D_H27Cα-F103Cα_ < D_H27Cα-S98Cα_.

The D_H27Cα–S98Cα_ and D_H27Cα–F103Cα_ time series gave insight into the pCdc25C-induced PRE changes for Ser^98^ and Phe^103^. Both residues showed strong PRE responses in the apo-PARA 3m-Pin1 sample and defined the α1/α2 contact region. Binding of pCdc256C to the WW domain reduced the Ser^98^ PRE, but not that of Phe^103^ (Fig. 5). The basis for this differential response became apparent from their differences, D_H27Cα–F103Cα_ − D_H27Cα–S98Cα_. Fig. 7*A* shows the time trace of these differences over the trajectory. Negative values indicate that Phe^103^ is closer to His^27^ (∼90% of the snapshots), whereas positive values indicate that Ser^98^ is closer. The fluctuations are due mainly to Ser^98^, given that D_H27Cα–S98Cα_ sampled a somewhat broader range of distances (∼74 Å) compared with D_H27Cα–F103Cα_ (∼63 Å). The time trace (Figure 6, Figure 7) shows that the differences between D_H27Cα–F103Cα_ and D_H27Cα–S98Cα_ can range from –10 to 5 Å. These fluctuations suggest how binding of the pCdc25C substrate could selectively reduce the PRE of Ser^98^, but not Phe^103^. Binding could stabilize conformations with the more extreme differences, such as the conformation at 610.6 ns, shown in Fig. 7*B*, which has S98Cα ∼10 Å further away from position 27 than Phe^103^. Because PRE line broadening is proportional to 〈1/*r*^6^〉, such differences could selectively reduce the Ser^98^ PRE. In this sense, conformations with these features could represent preexisting substrate-bound conformations. Therefore, it appears that the apo-Pin1 MD simulation not only samples interdomain contacts consistent with the apo-3m-Pin1 PREs, but also captures interdomain conformations that could account for the PREs of pCdC25C-bound state.

### Correlations between inter- and intradomain distances sensitive to substrate binding

Binding of the pCdc25C substrate induced changes of opposite sense in interdomain *versus* intradomain distances. Specifically, it decreased the PREs related to interdomain distances, indicating increased domain separation, while concomitantly increasing the PREs of some intra-WW domain distances, indicating some perturbation of the WW domain conformation.

This spurred our interest in whether the response might have its origins in the conformational ensemble of the apoprotein. Specifically, we considered the possibility that these opposite-sense changes might reflect correlations between interdomain and intradomain (WW) distance fluctuations.

We first investigated this possibility by calculating Pearson correlation coefficients (*r* values) between pairs of interdomain/intradomain distances (Table 1). The magnitudes (absolute values) of the correlation coefficients were rather modest. We regarded as significant only those coefficients with magnitudes ≥0.05. The 0.05 cutoff was based on the estimated S.E. and significance of the linear correlation coefficient (35, 36) (see “Experimental procedures”). Despite their modest magnitudes, correlation coefficient signs showed a striking consistency with the PREs. In particular, the coefficients between the interdomain distances and the intra-WW domain distances His^27^ Cα–Leu^7^ Cα and His^27^ Cα–Arg^14^ Cα were negative (anti-correlated), as would be expected from their opposite-sense PRE changes induced by pCdc25C binding. Also, the inter-/intradomain correlation coefficients involving the WW domain distance His^27^ Cα–Ala^31^ Cα were positive, consistent with the same-sense changes observed for Ala^31^ PREs upon pCdc25C binding. Specifically, the Ala^31^ cross-peak was completely broadened out in the apo-PARA 3m-Pin1 but reappeared in Cdc-PARA 3m-Pin1, indicating that pCdc25C binding increased the average Ala^31^–His^27^ distance.

**Table 1:** Pearson correlation coefficients between intradomain (rows) and interdomain (columns) distances Boldface type indicates correlation coefficients with magnitudes (absolute values) ≥ 0.05. This value is based on the estimated S.E. and quantitative significance of the correlation coefficient (35, 36) (see “Experimental Procedures”). —, correlation coefficients excluded by the 0.05 cutoff.

Intradomain WW	Interdomain distances			
	D_H27Ca–S98Ca_	D_H27Ca–D136Ca_	D_H27Ca–R142Ca_	D_H27Ca–H157Ca_
D_H27Ca–L7Ca_	**−0.05**	**−0.11**	**−0.09**	**−0.09**
D_H27Ca–R14Ca_	**−0.08**	**—**	**—**	**−0.05**
D_H27Ca–G39Ca_	**—**	**—**	**—**	**—**
D_H27Ca–S32Ca_	**—**	**—**	**—**	**—**
D_H27Ca–A31Ca_	**0.05**	**0.06**	**0.05**	**0.05**
D_H27Ca–F25Ca_	**—**	**—**	**—**	**—**

**Figure 8:**
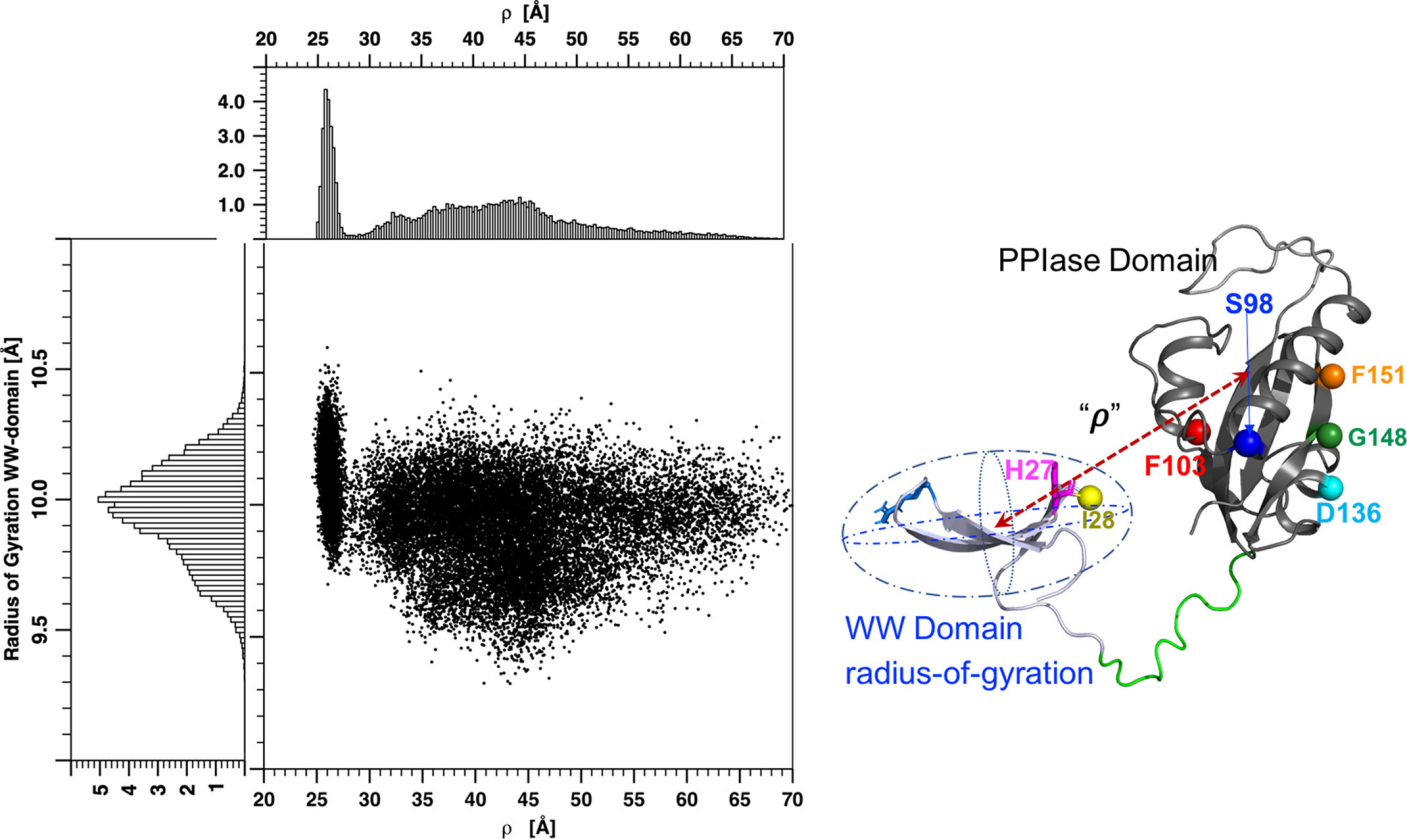
**Correlations between inter- and intradomain distance fluctuations.***Left*, scatter plot correlating interdomain separation (ρ) with the radius of gyration for the WW domain. Each *dot* is a snapshot from the apo-Pin1 MD simulation. The *horizontal histogram* refers to ρ, the distance between the domain centers of mass, schematized by the *red arrow* on the *right*. The *vertical axis* histogram refers to the WW domain radius of gyration and gives a measure of its compactness.

A known caveat of the Pearson correlation coefficient is the assumption of a *linear* relationship between two quantities. Consequently, low-magnitude correlation coefficients may indicate a lack of correlation, a nonlinear relationship, or both. Acknowledging this, we explored the relationship between the interdomain/intradomain distances visually, using the scatter plot in Fig. 8 Here, we examined two diagnostic distances, including the distance between the domain centers-of-mass (denoted as ρ, *horizontal axis*), and the WW domain radius of gyration (the square root of the trace of eigenvalues for the WW domain gyration tensor, *vertical axis*). Each *dot* in Fig. 8 represents an MD snapshot. The histograms on the *axes* represent marginal distributions. The WW domain radius of gyration serves as a measure of intradomain distance, or compactness, whereas ρ is a generalized interdomain distance. If interdomain and intradomain distances were completely uncorrelated, then Fig. 8 should reflect the simple products of their separate probability distributions. Fig. 8 shows this is not the case: shorter domain separations show a preference for more extended WW domain conformations, whereas larger separations prefer more compact WW domain conformations. These preferences suggest correlations between their fluctuations. Such correlations are consistent with the PREs whereby upon pCdc25C binding, the interdomain distance increased, whereas the intra-WW domain distances, D_H27Cα–L7Cα_ and D_H27Cα–R14Cα_, decreased, signifying increased compaction (increased concavity) of the WW domain (see above).

### Correlations between interdomain distances and intradomain interresidue contacts

We explored the influence of interdomain separation on another class of metrics sensitive to intradomain conformation: interresidue contact numbers. For an arbitrary pair of residues, the interresidue contact number is the number of heavy atom pairs (one atom from each residue) within 4.5 Å of each other (37). As the protein conformation fluctuates during the MD trajectory, so do the interresidue contact numbers. For a given pair of residues, the fluctuating contact number can be extracted as a time series from the trajectory and then correlated with other time series, such as the interdomain distances highlighted by the PREs.

We identified 1386 residue pairs with fluctuating contact numbers: 279 in the WW domain and 1107 in the PPIase domain. We then calculated the Pearson correlation coefficients between the time series of the 1386 residue pairs with each of the four interdomain distances most sensitive to pCdc25C binding: D_H27Cα–S98Cα_, D_H27Cα–D136Cα_, D_H27Cα–R142Cα_, and D_H27Cα–H157Cα_. Thus, associated with each of the four distances was a pool of 1386 correlation coefficients, each referring to a particular interresidue contact.

**Figure 9:**
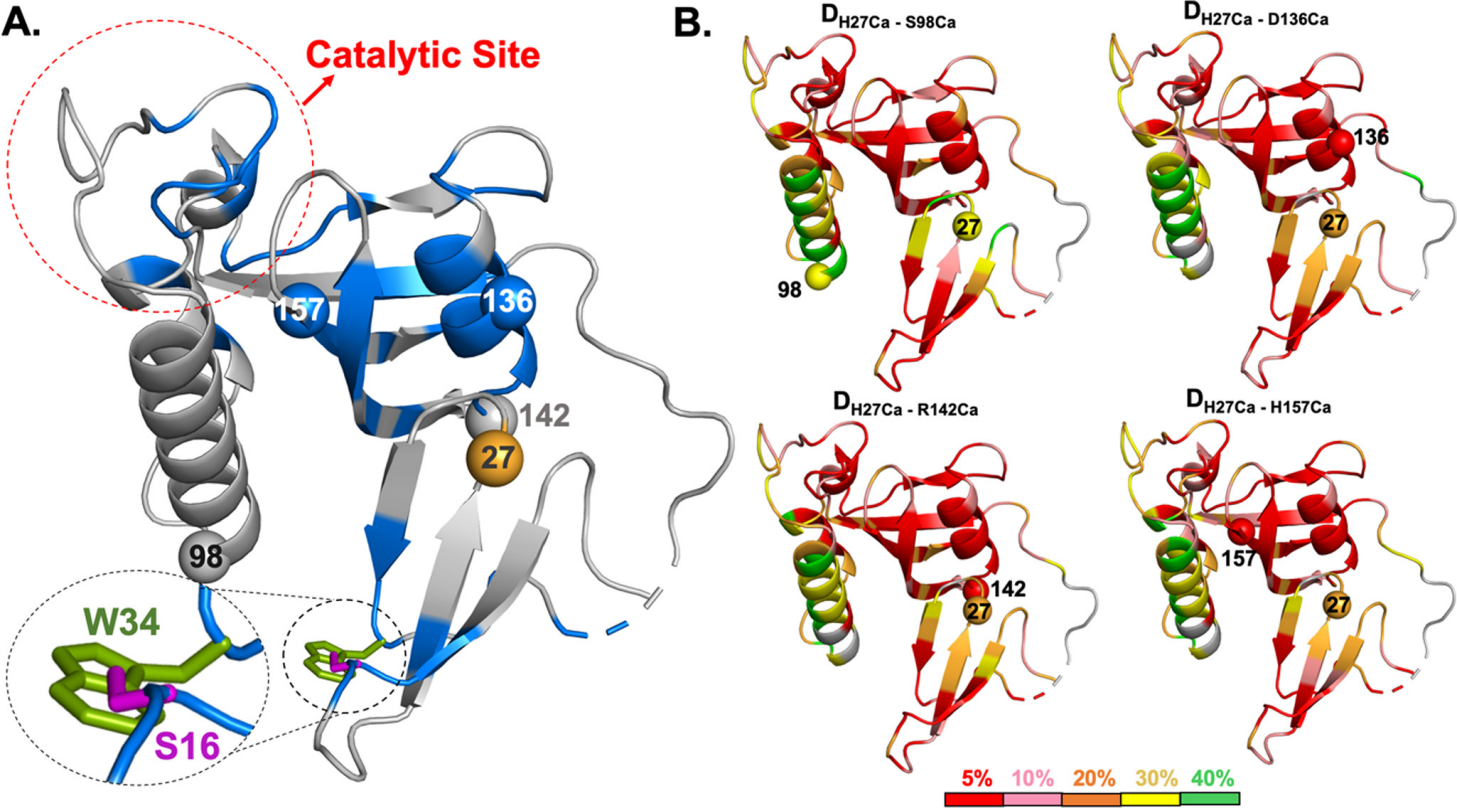
**Pairwise intradomain residue contacts that correlate with different interdomain distances.***A*, *blue shading* denotes residues engaged in pairwise contacts showing the largest-magnitude correlation coefficients (the top 5%) with the PRE-identified interdomain distances (D_H27Ca-S98Ca_, D_H27Ca-D136Ca_, D_H27Ca-R142Ca_, and D_H27Ca-H157Ca_). The *top dashed oval* denotes PPIase residues important for isomerase activity; the *bottom dashed ovals* highlight WW domain residues Ser^16^ and Trp^34^ that mediate substrate binding. *B*, *color-coded depiction* of contact/distance correlation coefficients. Coefficient magnitudes within the top 5, 10, 20, 30, and 40% are *red*, *pink*, *orange*, *yellow*, and *green*, respectively. Thus, *red* denotes the largest-magnitude correlation, whereas *green* indicates the lowest. The *red shading* reveals apparent “passageways” linking the WW domain substrate-binding site and the distal PPIase active site, for each of the four interdomain distances.

Then for each of the four distances, we identified the correlation coefficients with the largest magnitude (top 5%) and their associated interresidue pairs (contacts). The top 5% includes correlation coefficients with magnitudes in the top 2.5% of the positive and negative coefficients (Table 2). (The distribution of correlation coefficients specific for each interdomain distance are given as histograms in Fig. S4). The pairwise residue contacts of high correlation *common* to all four interdomain distances are displayed in Fig. 9*A*. They reside predominantly in β-sheet regions in both domains. They also coincide with subregions supporting substrate binding and catalysis, such as the catalytic pocket of the PPIase domain and the substrate-binding site of the WW domain defined by Trp^34^ and Loop 1 residues Ser^16^–Arg^21^. These highlighted locations suggest that changes in interdomain distance perturb the domain regions contributing to the functional mechanism of Pin1.

**Table 2:** Correlation between interresidue contact numbers and interdomain distances Boldface type indicates contacts between residues at least 3 residues apart in the amino acid sequence. Italic type indicates contacts involving Trp^34^ in the WW domain. —, correlations failing to meet the 5% histogram cutoff.

	D_H27Ca–S98Ca_	D_H27Ca–D136Ca_	D_H27Ca–R142Ca_	D_H27Ca–H157Ca_
**C_R14–Y23_**	**−0.38**	**−0.24**	**−0.28**	**−0.29**
**C_R14–V22_**	**-0.25**	**—**	**—**	**—**
*C_Q33_*_–_*_W34_*	−*0.23*	−*0.23*	−*0.23*	−*0.24*
C_R21–V22_	−0.19	—	—	—
C_K13–M15_	−0.17	−0.19	−0.21	—
**C_D3–K6_**	**—**	**—**	**—**	**−0.23**
C_G20–V22_	0.19	0.20	—	—
***C_V22_*_–_*_W34_***	***0.20***	**—**	**—**	**—**
**C_R21–E35_**	**0.21**	**—**	**—**	**—**
**C_V22–Q33_**	**0.21**	**—**	**—**	**—**
***C_S19_*_–_*_W34_***	***0.23***	**—**	**—**	**—**
C_M15–R17_	0.23	0.22	0.24	0.23
C_V22–Y24_	0.25	—	—	—
C_S19–R21_	0.25	—	—	—
C_R14–S16_	0.25	—	—	0.20
**C_Y23–Q33_**	**0.26**	**0.19**	**0.23**	**0.22**
C_P37–G39_	0.27	0.27	0.30	0.27
**C_Y23–S32_**	**0.29**	**0.25**	**0.29**	**0.27**
**C_V22–E35_**	**0.30**	**—**	**—**	**—**
**C_S16–Y23_**	**0.35**	**0.28**	**0.33**	**0.31**
***C_S16_*_–_*_W34_***	***0.35***	***0.27***	***0.33***	***0.30***

The four structures in Fig. 9*B* provide “spatially resolved” maps of higher *versus* lower magnitude correlations for each of the four interdomain distances. The WW domain shows a persistent pattern: maximal correlation coefficients localize to the substrate binding Loop 1 and Trp^34^ and then attenuate in the direction of Loop 2 at the other end of the domain. The attenuation smacks of “signal decay” similar to that observed in our previous study of the Pin1 WW domain with a destabilizing substitution mutation Q33E (38). In that study, the attenuation direction of Q33E-induced CSPs was perpendicular to the β-sheet strands, indicating weakened cross-strand hydrogen bonds important for thermal stability. Here, the attenuation of distance-contact correlations runs parallel to the β-sheet, consistent with the established “functional gradient” of the Pin1 WW domain (Loop 1 and Trp^34^ at one end mediates substrate binding, whereas Loop 2 at the other end mediates transient contacts with the PPIase domain). Fig. 9*B* and Table 2 also show some subtle differences in the spatial distribution of the high-correlation contacts for the four interdomain distances. We return to this point under “Discussion.”

### Modulation of WW domain conformation accompanies interdomain distance changes

**Figure 10:**
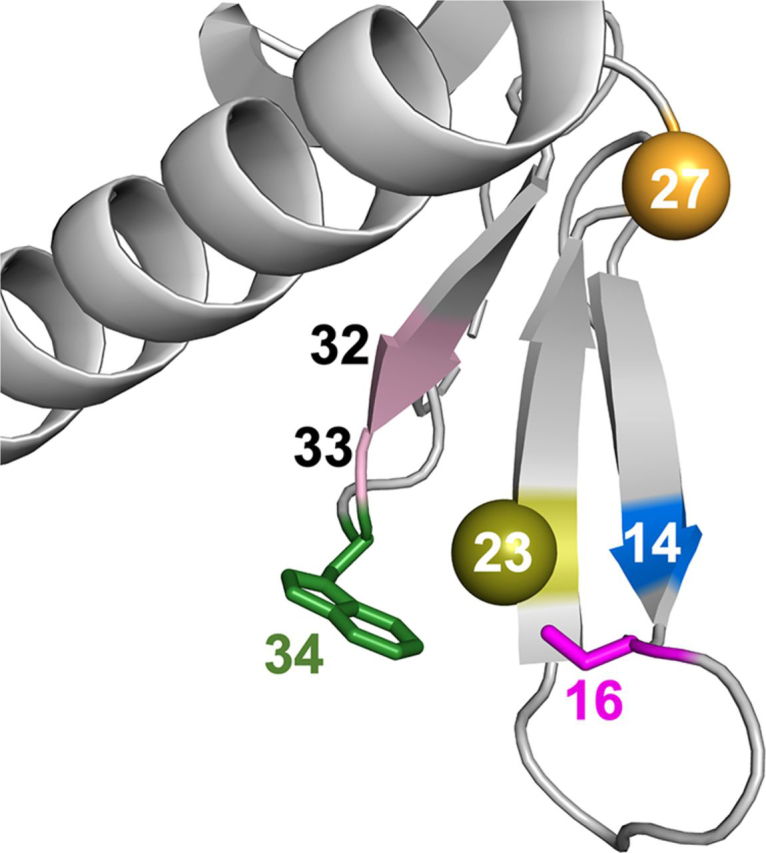
**Markers of WW domain conformation correlating with interdomain distance.** Increased interdomain distances are accompanied by weaker contact of C_R14–Y23_ and stronger contacts of C_Y23–Q33_, C_Y23–S32_, C_S16–Y23_, and C_S16–W34_.

We wanted to explore the significance of the interresidue contacts described above to WW domain conformation. Fig. 10 focuses on the high-correlation interresidue contacts (top 5%) in the WW domain that are common to all four interdomain distances. Most cluster around Tyr^23^, a residue important for substrate recognition (29). Notable contacts included C_Y23–R14_(−), C_Y23–S16_(+), C_Y23–S32_(+), C_Y23–Q33_(+), C_Y23–S16_(+), and C_S16–W34_(+) (Table 2), where the parenthetical signs are the signs of the correlation coefficients. Except for C_Y23–R14_, all such contacts had positive correlation coefficients, indicating an increase of intradomain contacts upon an increase of interdomain distance. Increased contact suggests local compaction. The exception is C_Y23–R14_(−) (Fig. 10), the only negative correlation coefficient indicating loss of contact.

The central location of Tyr^23^ in the WW domain stands out for two reasons. First, the X-ray crystal structure of Pin1 complexed with the doubly phosphorylated peptide representing the C-terminal domain of RNA polymerase II by Verdecia *et al.* (29) identified it as an internal pivot point for the conformational changes needed to bind phosphopeptide substrate. Remarkably, our apo-state simulations sampled interresidue contact changes around Tyr^23^ consistent with those needed for substrate binding and further revealed their correlation with changes in interdomain distances. Second, Tyr^23^ is part of a conserved hydrophobic core II; thus, its “pivot” function may be a defining feature of the WW domain family.

### Two-cluster model

The distance-contact analysis above suggests that interdomain proximity influences the conformations sampled by the WW domain. This raises the possibility that apo-Pin1 can exchange between at least two conformational subensembles: one with conformations compatible with a more “compact” Pin1 (proximal domains) and another with conformations compatible with an overall extended Pin1 (distal domains). We previously discussed such exchange as part of a speculative model to explain Pin1 interactions with substrate having multiple pS/T-P sites (13).

To investigate this possibility, we clustered the 22,440 MD snapshots based on the interdomain distance His^27^ Cα–Ser^98^ Cα_,_ the distance showing the greatest quantifiable change in PRE (decrease) upon pCdc25C binding. We used the average-linkage approach in the CPPTRAJ program (39), which produced two clusters of Pin1 conformations, designated Cluster_COMPACT_ and Cluster_EXTENDED_, with average D_H27Ca–S98Ca_ values of 32.7 and 50.1 Å, respectively.

We assessed the merit of these two clusters by checking their abilities to reproduce the sensitivity of interresidue contacts to interdomain separation (Fig. S5). Indeed, Cluster_EXTENDED_ (distal PPIase and WW domains) displayed more intimate contacts of C_S16–W34_, C_S16–Y23_, C_Y23–Q33_, and C_Y23–S32_, and a weaker contact of C_R14–Y23_, relative to Cluster_COMPACT_ (proximal PPIase and WW domains).

**Figure 11:**
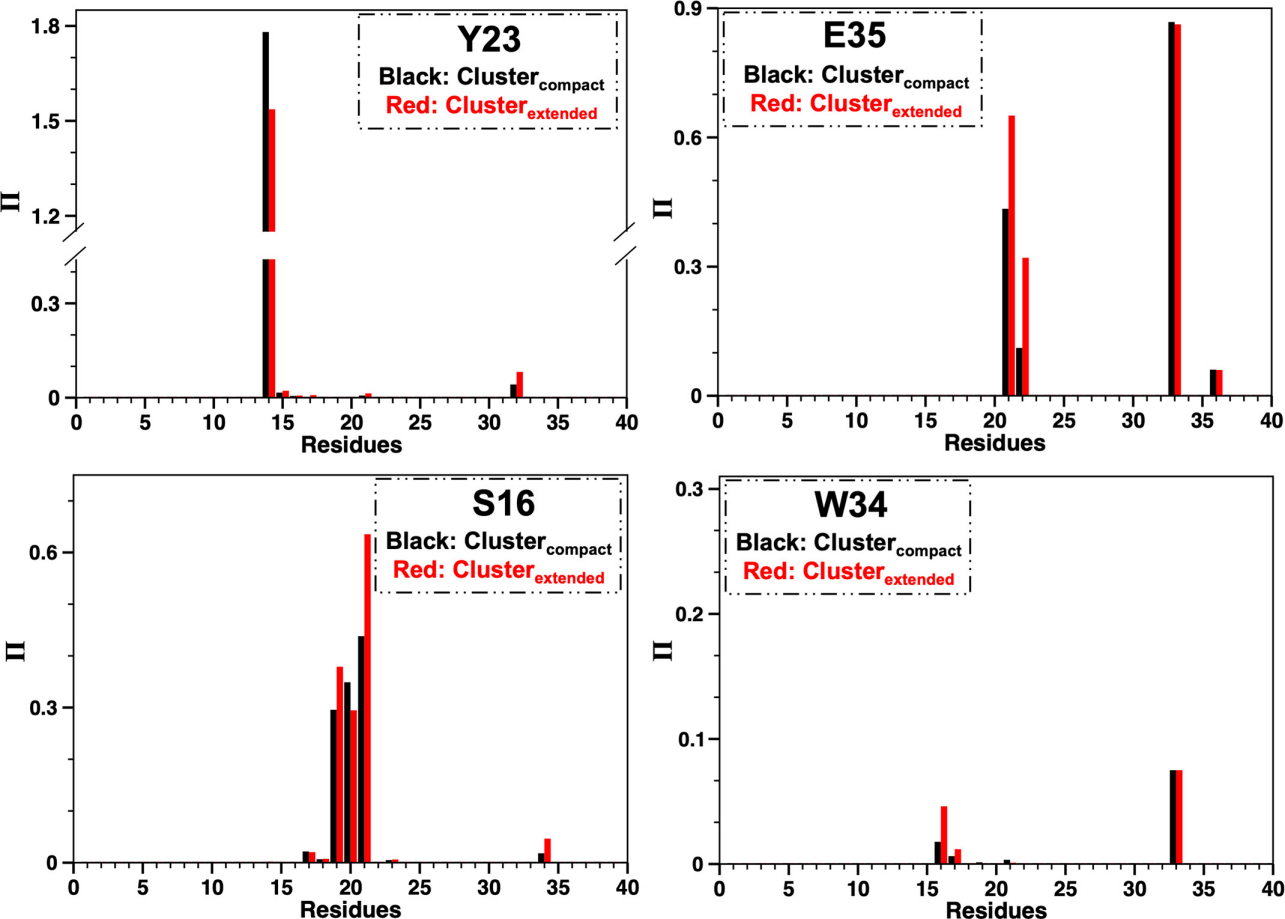
**Response of H-bonds to local conformational changes in the WW domain.** The average occupancy H-bonds involving Tyr^23^, Glu^35^, Ser^16^, and Trp^34^ in the two clusters corresponding to the compact (*black*) and extended (*red*) form of Pin1.

To understand the atomic basis of the conformational change in the WW domain, we then compared the hydrogen bond patterns of the two clusters. Our metric was the sum over average occupancies of hydrogen bonds between residue pairs (see “Experimental procedures”), as in our previous work (38). We found that the average hydrogen bond occupancy in Cluster_EXTENDED_ *versus* Cluster_COMPACT_ mirrored the aforementioned changes in interresidue contact numbers (Fig. 11). Specifically, in Cluster_EXTENDED_, a weaker H-bond_R14-Y23_ coincided with weaker contact between Arg^14^ and Tyr^23^, whereas the increased H-bond occupancies of H-bond_Y23–S32_, H-bond_R21–E35_, H-bond_V22–E35_, and H-bond_S16–R21_ collectively brought Trp^34^ closer to Loop 1 (Ser^16^–Arg^21^), creating a more compact substrate-binding site.

## Discussion

The design of Pin1 illustrates a strategy common among eukaryotic signaling proteins: a single chain folded into discrete domain modules connected by flexible linkers (1, 2). Linker flexibility allows for relative domain motion that could influence the interdomain contacts supporting function. For example, Pin1 has numerous protein substrates; possibly, interdomain flexibility helps Pin1 adapt to the conformational diversity presented by its varied substrates, which include both tumor suppressors and oncogenes (40).

The pCdc25C phosphopeptide substrate studied in this work preferentially binds at the WW domain substrate-binding pocket (Trp^34^ and Loop 1: Ser^16^–Arg^21^) (41). Our previous NMR studies showed that such binding reduces interdomain contact relative to the apo-state and alters *cis*-*trans*-isomerase activity (13). However, some important questions remained outstanding. First, which residues mediate the transient WW/PPIase domain contacts in its apo-state? Second, *how* does substrate binding weaken those interdomain contacts? In principle, weaker domain contact reflects an increase of rotational mobility of one domain relative to another, an increase in domain separation, or both. More direct probes of the distance effects have therefore been wanting. The PRE measurements and MD simulations are examples of such probes; they have given us several new insights that answer some of the above questions.

First, the PREs of apo-3m-Pin1 have revealed a new region of transient interdomain contact in the α1/α2 region of the PPIase domain. These PREs expand the domain interaction surface beyond the α4/β6/β7 region suggested by previous Pin1 crystal structures (15, 29) and NMR chemical shift perturbations (11, 13). These results suggest that apo-Pin1 samples a range of proximal domain configurations. Conceivably, this could promote its ability to access and bind a diverse range of protein substrates. Sampling multiple “compact” configurations could also reduce the loss of conformational multiplicity (smaller entropic penalty) when transitioning from extended domain configurations to more compact ones as seen upon binding of some Pin1 substrates.

Second, comparisons of the PREs from the apo- and pCdc25C-complexed 3m-Pin1 samples revealed an increase in the average interdomain distances between WW domain Loop 2 and the entire PPIase domain–interacting surface. These results unequivocally demonstrate that the loss of transient interdomain contact upon pCdc25C binding to the WW domain includes an increase of domain separation, and not merely more vigorous rotational mobility.

Third, the binding-induced changes in the PREs also included decreases of certain intradomain distances within the WW domain. Thus, the binding-induced changes in interdomain conformation (increased separation between the do-mains) are coupled to changes of intradomain conformation that affect local compaction. Such PRE changes are experimental signs of correlations between inter- and intradomain motion.

It is well-appreciated that substrate binding by single-domain proteins can involve “conformational selection,” whereby an incoming substrate binds to and stabilizes a subset of preexisting apo-state conformers. In this process, correlated conformational fluctuations within the apo-domain give rise to conformations resembling that of the bound substrate. The Pin1 PREs suggest that we can extend this notion to interdomain degrees of freedom characteristic of multidomain proteins. In other words, correlated fluctuations among the interdomain and intradomain degrees of freedom give rise to multidomain conformations resembling that of the bound substrate.

The MD trajectory of apo-Pin1 let us explore this hypothesis. For example, PREs highlighted four interdomain distances, D_H27Ca–S98Ca_, D_H27Ca–D136Ca_, D_H27Ca–R142Ca_, and D_H27Ca–H157Ca_, showing the greatest increases upon pCdc25C binding. We calculated Pearson correlation coefficients between these distances and intradomain distances for 22,400 MD snapshots. Whereas the coefficient magnitudes were modest, their signs were consistent with the PRE changes induced by pCdc25C binding, namely compaction of the WW domain concomitant with increased interdomain distances. These correlations are supported by the scatter plot of Fig. 8, which shows a preference for more compact WW domain conformations (smaller radii of gyration) at greater interdomain separation (greater ρ values). Furthermore, correlations between interdomain distances and intra-WW domain contacts (Figure 9, Figure 10) also suggest a more compact substrate-binding site in the WW domain when the interdomain distances increase. Hence, the apo-Pin1 MD simulation suggests that correlated conformational fluctuations include the conformational changes that facilitate pCdc25C binding.

The intradomain residue pairs with the largest-magnitude correlations (between intradomain contacts and interdomain distances) overlapped significantly for the four interdomain distances, an unsurprising result considering that the distance fluctuations were not independent. As shown in Fig. 9*A*, common residue pairs occur in the WW domain substrate-binding site (Trp^34^ and Loop 1) and within the catalytic site of the PPIase domain. These locations bolster the notion of cross-talk between these distal sites via internal “synchronization”: loss or gain of interdomain contact. These contact changes at the PPIase domain surface propagate to the hydrophobic pocket for PPIase activity by local changes in side-chain flexibility highlighted by the dynamic conduit noted in previous side-chain dynamics studies (19).

As noted above, the four interdomain distances also show some variation in the spatial distribution of their high-correlation interresidue contacts (*cf*. Table 2 and Fig. 9*B*). Such variation raises the possibility of multiple, overlapping “passageways” connecting the WW domain substrate-binding site to the distal PPIase active site. These “passageways” are related to the dynamic “conduit” we had proposed earlier (19, 42) as a mechanism for allosteric communication between the PPIase α4/β6/β7 residues available for interdomain contact and residues in the PPIase active-site pocket. Conceivably, different interdomain conformations could be induced via different sets of *intra*domain conformational changes upon recognition of distinct substrates. The scenario is attractive when trying to explain the broad range of Pin1 substrates, which could enhance or decrease the interdomain contacts.

Some caveats of our simulation analysis deserve comment. First, the simulations suggest potentially long dwell times for interdomain association. A clear example is the stable segment of closer contact, 0.9–1.8 ns in Figure 6, Figure 7. This suggests that a proper weighting of domain configurations for quantitative comparisons with the experimental PREs would need even longer sampling. A practical way to pursue this could exploit alternative simulation methods better suited for large-scale motions, such as Map-SGLD-NMR (43). Second, the low Pearson correlation coefficients are explained, at least in part. Specifically, the Pearson coefficients assume a linear relationship between the two fluctuating quantities, and they can take on low values when the prevailing relationship is nonlinear, as indicated by the shape of Fig. 8. Alternative methods for correlated motion analysis of MD simulations are available that bypass the assumption of linearity, such as those based on mutual information (44, 45). Work is in progress to use these methods to explore the correlated motion indicated by our PRE data.

A core premise of this work is a substrate like pCdc25C that preferentially binds the WW domain, thereby weakening the apo-state level of transient interdomain contact. This is not overly restrictive; preferential binding to the WW domain is thought to be common among biological Pin1 substrates, which correspond to pS/T-P sequences within disordered segments of other cell-signaling proteins. Thus, the coupling of inter-/intradomain distance fluctuations revealed by pCdc25C is likely relevant for many other Pin1 substrates.

**Figure 12:**
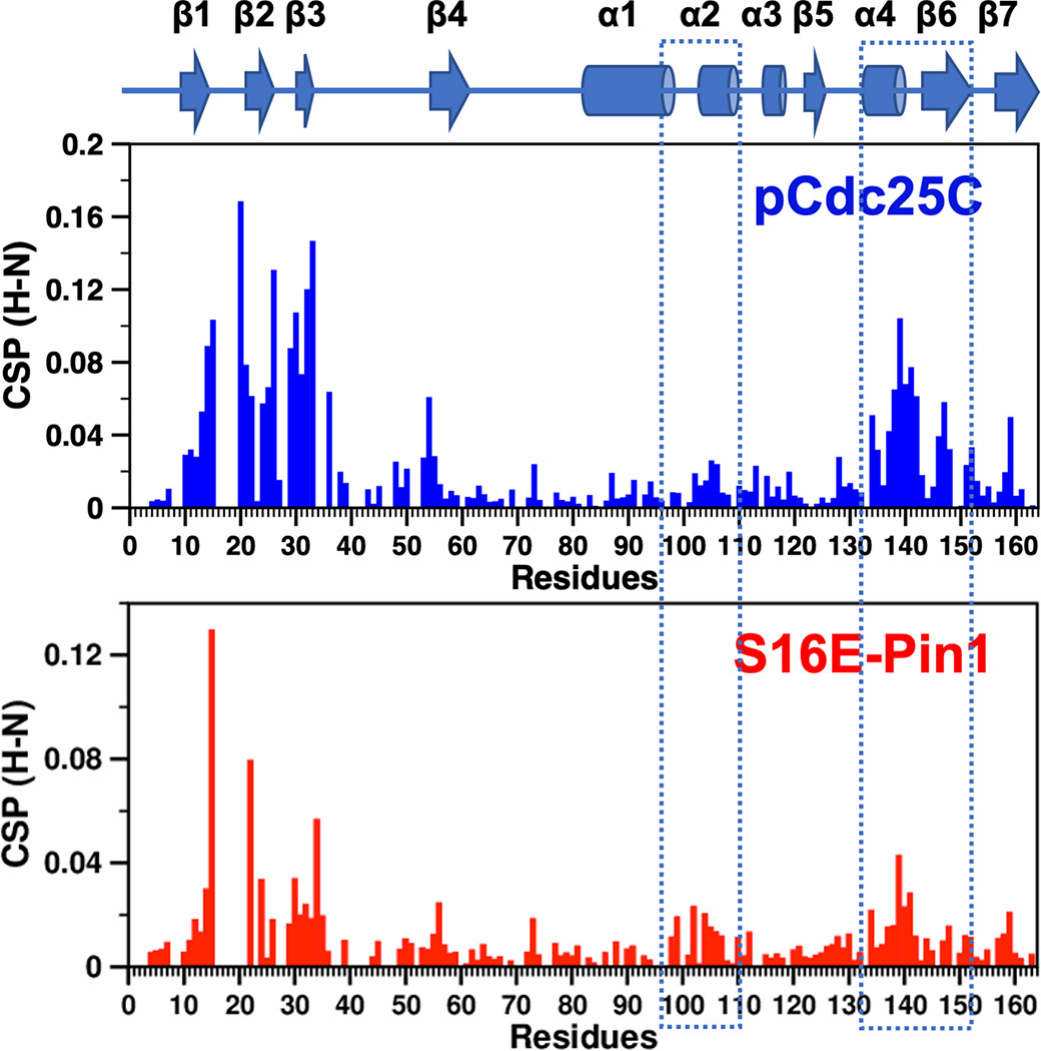
**Backbone NH CSPs from different perturbations to the WW domain.***Top*, pCdc25C binding to the WW domain; the CSPs reflect pCdc25C-complexed WT-Pin1 *versus* apo-Pin1. *Bottom*, S16E substitution to mimic phosphorylated Ser^16^; the CSPs reflect apo-S16E-Pin1 *versus* apo-WT Pin1. NH CSP surges in PPIase regions for interdomain contact are prominent in both cases (*dotted rectangles*).

Finally, we discuss the potential significance of these findings to other types of WW domain perturbations. In other words, the reduced interdomain contact may be a result of a broader range of WW domain perturbations besides substrate binding. These would include Pin1 post-translational modifications of the Pin1 WW domain, such as SUMOylation (14) and phosphorylation (10). In the latter case, Pin1 has several serines for which post-translational phosphorylation changes isomerase activity, subcellular location, or susceptibility to proteasomal degradation (6, 10, 46, 47). For example, post-translational phosphorylation of Ser^16^ (pS16) by protein kinase A inhibits substrate binding and nuclear localization (10), but the atomic-level consequences of this phosphorylation event remain unclear. Notably, pS16 introduces a negative charge to the same Loop 1 region of the WW domain as pCdc25C. Could the mechanism for Ser^16^ post-translational phosphorylation involve a similar mechanism of domain cross-talk as shown by pCdc25C binding? To begin answering this question, we have generated S16E-Pin1, a mimic of pS16 also used in cell assays (10). An analysis of S163E-Pin1 backbone ^1^H-^15^N CSPs (apo-S16E *versus* apo-WT-Pin1) reveals a response resembling pCdc25C binding–chemical shift perturbations to PPIase residues in the α4/β6/β7 region contacting the WW domain (Fig. 12). We expect further experiments will show a similar, yet distinct response, given that pS16 (or the S16E substitution) is a localized perturbation compared with the binding of a 10-residue phosphopeptide; hence, the effects on domain contact might be smaller.

## Conclusions

Our PRE experiments show that the transient interdomain contacts in apo-Pin1 exceed the range previously suggested. PREs also show reduced interdomain contact upon binding of pCdc25C to the WW domain, which involves increased domain separation concomitant with intra-WW domain conformational shifts consistent with those induced by binding of pS/T-P substrates (13, 29, 30). Our corresponding 4.5-μs MD simulation of apo-Pin1 suggests that these substrate-induced changes may preexist as rare, correlated fluctuations in the apo-Pin1 ensemble. This widens the scope of the conformational selection model to include interdomain/intradomain correlations, with substrate binding stabilizing preexisting subconformations inherent in the apoprotein. The correlation coefficients between 1386 intradomain contacts and four interdomain distances raise the possibility of multiple, overlapping atomic “passageways” or “conduits” linking the distal WW substrate binding and PPIase catalytic sites. Presumably, different interdomain conformations could be induced by different intradomain conformational changes initiated by the binding of distinct substrates or post-translational modifications. Internal dynamics enabling an adaptive response to different conformational perturbations could explain the broad range of Pin1 substrates and its varied responses to post-translational modifications. Many signaling proteins share the dynamic modular architecture of Pin1; hence, the inter-/intradomain coupling indicated here may be a common mechanism.

## Experimental procedures

### Overexpression and purification of 3m-Pin1 and WT-Pin1

For specific spin labeling at residue 27, we generated the triple-mutation construct, H27C/C57S/C113D-Pin1 (3m-Pin1). The C57S and C113D substitutions were to prevent off-target spin labeling. Cys^57^ is largely surface-exposed, and the choice of C57S was based on side-chain similarity. Cys^113^ is part of the substrate proline-binding pocket. We chose C113D based on previous studies demonstrating that C113D-Pin1 maintains activity *in vivo* and *in vitro* (29).

Both H27C/C57S/C113D-Pin1 (3m-Pin1) and WT-Pin1 were overexpressed in *Escherichia coli* BL21 (DE3) cells (Novagen). Cells were first grown at 37 °C in lysogeny broth medium until they reached an *A*_600_ of 0.8–1.0. For isotope-enriched protein, cells were harvested and resuspended in M9 minimal medium containing ^15^NH_4_Cl and/or [^13^C]glucose (Cambridge Isotope Laboratories) as the sole nitrogen and carbon sources (48). Overexpression of 3m-Pin1 was induced by adding 1 mm isopropyl β-d-1-thiogalactopyranoside and incubated at 16 °C (to slow expression and allow proper folding) for ∼20 h. Overexpression of WT-Pin1 was induced at 26 °C for ∼16 h. Cells expressing protein were harvested and resuspended in 50 mm HEPES buffer (pH 7.5 for WT-Pin1 and pH 6.5 for 3m-Pin1 containing 1 mm EDTA). Both 3m- and WT-Pin1 constructs were purified using a HiTrap SP column followed by size exclusion (HiPrep Sephacryl S-200 HR).

### Paramagnetic and diamagnetic moieties

Our paramagnetic spin label was MTSL (1-oxyl-2,2,5,5-tetramethyl-Δ^3^-pyrroline-3-methyl). Protein was eluted in buffer containing 50 mm sodium phosphate and 300 mm NaCl (pH 6.9) during size exclusion. DTT was added to a molar ratio of DTT/protein = 3:1. Paramagnetic MTSL was dissolved in ethanol and then added to the protein sample to a final molar ratio of MTSL/protein = 30: 1. The mixture was incubated overnight at 4 °C in the dark. Excess (free) label was removed using size-exclusion chromatography (avoiding light). Attachment of diamagnetic acetyl-MTSL was identical except that it did not require dark conditions. NMR samples were exchanged into 30 mm imidazole-*d*_4_ (Cambridge Isotope Laboratories) buffer (pH 6.6) containing 30 mm NaCl, 0.03% NaN_3_, 5 mm DTT-*d*_10_, and 90% H_2_O, 10% D_2_O using a 10,000 molecular weight cutoff centrifugal filter.

Notably, 3m-Pin1 tended to aggregate at a high concentration at a high temperature. Keeping the protein at low concentration and low temperature was critical during purification and labeling. The highest NMR sample concentration for 3m-Pin1 was ∼100 μm.

### Sequential NMR resonance assignments and chemical shift analysis

All 3m-Pin1 spectra were recorded on a Bruker Avance I spectrometer at 16.4 T (700.13 MHz ^1^H frequency) equipped with a TCI cryogenic probe (Bruker Biospin, Inc.). Sample concentrations ranged from 80 to 100 μm. The 3m-pin1 backbone assignments were confirmed using established three-dimensional HNCACB (49), HNCOCACB (50), and 2D ^1^H-^15^N HSQC (51) experiments at a nominal temperature of 295 K and comparisons with the WT-Pin1 assignments. NMR data processing used TopSpin 3.5 (Bruker Biospin) and resonance assignments made with Sparky 3 (52) and CARA (53). Amide ^1^H-^15^N CSPs were calculated using Equation 1,

(Eq. 1) $${\Delta \delta }_{\text{NH}}=\sqrt{{\left(\Delta \delta_{\text{H}}\right)}^{\text{2}}+{\left(\text{0.154}{\Delta \delta }_{\text{N}}\right)}^{\text{2}}}$$

where Δδ_H_ and Δδ_N_ are ^1^H and ^15^N chemical shift differences, respectively.

### Amide ^1^H paramagnetic relaxation enhancements

PRE rates refer to the enhanced spin relaxation rates of a nuclear spin due to its proximity to an unpaired electron. This study focused on the transverse PRE rate constant (Γ_2_). For a nitroxide spin radical like MTSL, the PREs are dominated by direct dipole-dipole interactions per the Solomon–Bloembergen expressions (20, 21). The transverse relaxation contribution is as follows (cgs units),

(Eq. 2) $$\Gamma_{2}=\frac{S(S+1){\left(g\mu_{B}\gamma_{I}\right)}^{2}}{15\langle {r_{\text{IS}}}^{6}\rangle }\left\{4\tau_{c}+\frac{3\tau_{c}}{1+{\left(\omega_{H}\tau_{c}\right)}^{2}}\right\}$$

where τ*_c_* reflects both overall rotational diffusion of the protein (τ*_R_*) and the effective electron relaxation time (τ_elec_) (20).

(Eq. 3) $$\frac{1}{\tau_{c}}=\frac{1}{\tau_{R}}+\frac{1}{\tau_{\text{elec}}}$$

The sum above assumes that electron relaxation is uncoupled from isotropic molecular tumbling.

In Equation 2, *r*_IS_ refers to the distance between an amide proton and the unpaired electron of the spin label (approximately at the nitrogen position of MTSL). The symbol µ*_B_* is the magnetic moment of the free electron (Bohr magneton), *S* is the electron spin quantum number, *g* is the electron *g* factor, γ*_Ι_* is the gyromagnetic ratio of the amide proton, and ω*_Η_* is its Larmor frequency. Equation 2 assumes that dipole-dipole interaction vectors are well-approximated as rigid in the molecular frame on the time scale of overall molecular tumbling. More complex expressions can be used for rigorous incorporation of rapid internal motion (23).

The PREs (Γ_2_(^1^H^N^) values) are proportional to the ensemble average of the inverse sixth power of the interspin distance (*i.e.* 〈r_IS_^−6^〉), where the unpaired electron spin location is approximated by the nitrogen of the nitroxide spin label. Under the reasonable assumption that domain reorientational and translational motions are rapid on the chemical shift time scale, we can semiquantitatively interpret the experimental PREs of different amide protons as indicative of their relative proximity to the paramagnetic label (22). The PREs were taken as the difference of the amide proton transverse relaxation rate constants, Γ_2_(^1^H^N^) = *R*_2,PARA_(^1^H^N^) − *R*_2, DIA_(^1^H^N^). The latter were measured using established 2D ^15^N-^1^H pulse schemes (24) with relaxation delays of 4 (2×), 6, 8, 10, 12, 15, 20 (2×), 25, and 30 ms, where “2×” indicates duplicate measurements.

The threshold values were determined by the sum or difference of the mean and double of the S.D. of the twice-filtered PREs. Specifically, we calculated the mean (M1) and S.D. (STD1) of all PREs and filtered PREs falling outside of M1 ± STD1; we then calculated the mean (M2) and S.D. (STD2) of the remaining PREs and likewise filtered PREs falling outside of M2 ± STD2. The remaining PREs after the second filter were taken as the core values. We further calculated the mean (M3) and S.D. (STD3) of the core PREs and defined the threshold value as M3 ± 2·STD3.

### ^15^N relaxation rate constants

Backbone ^15^N spin relaxation rate constants (*e.g.* R_1_(N), R_2_(N)) report on the spectral density functions *J*(ω) describing the rotational dynamics of ^15^N–^1^H bond vectors relative to the laboratory static magnetic field. For slowly tumbling molecules such as proteins at high magnetic field strengths, the combination of rate constants ^15^N *R*_2_-*R*_1_/2 is approximately the following (54),

(Eq. 4) $$R_{2}(N)-\frac{R_{1}(N)}{2}=\frac{2C_{N}}{3}\left(1+\frac{3D_{IN}}{C_{N}}\right)J_{\text{eff}}(0)$$

where

(Eq. 5) $$C_{N}=\frac{\Delta_{N}^{2}}{3}$$

and

(Eq. 6) $$D_{IN}=\frac{\hbar^{2}\gamma_{I}^{2}\gamma_{N}^{2}}{\langle r_{NH}^{6}\rangle }$$

*D_IN_* refers to the ^15^N-^1^H heteronuclear dipole-dipole interaction, and *C_N_* reflects the anisotropy of the ^15^N chemical shielding tensor. In the absence of chemical exchange processes and assuming isotropic overall tumbling,

(Eq. 7) $$J_{\text{eff}}(0)=J(0)=\frac{2\tau_{c}}{5}$$

where τ_c_ is the effective rotational correlation time of the NH bond.

We measured the backbone amide ^15^N *R*_2_-*R*_1_/2 for apo-DIA 3m-Pin1, Cdc-DIA 3m-Pin1, apo-WT-Pin1, and Cdc WT-Pin1, using a consolidated 2D ^15^N-^1^H pulse scheme. The relaxation delays included 4.12 (2×), 8.24 (2× for Cdc-DIA 3m-Pin1), 12.36, 16.48, 24.72, 28.84, 32.96, 37.08, and 41.2 ms (2× for apo-DIA 3m-Pin1, apo-WT-Pin1, and Cdc WT-Pin1). Cross-peak intensity *versus* relaxation delay were fitted to monoexponential decays with *R*_2_-*R*_1_/2 as one of the parameters. Uncertainties were estimated using Monte Carlo simulations with noise estimates from the duplicate spectra.

### Explicit solvent MD

All-atom MD simulations of WT-Pin1 were performed at 300 K using the GPU (CUDA) version (55, 56, 57) of the AMBER 16 software package (PMEMD) (31) with ff14SB force field (58) and the “optimal” three-charge, four-point rigid water model (OPC) (32). The first model of the WT-Pin1 NMR structure (PDB entry 1NMV) (17) was used as the starting structure of WT-Pin1.

After energy minimization, the system underwent three steps of equilibration (0.8, 8, and 80 ns) with positional restraint factors of 10, 1, and 0.1 kcal·(mol Å^2^)^−1^ respectively. Prior to the production run, we implemented hydrogen mass repartitioning (59) to allow for a longer time step (4 fs) in the production runs (∼4.5 μs for apo-WT-Pin1). We generated an NTP ensemble, using a Langevin thermostat with a collision frequency of 5 ps^−1^ and a Berendsen barostat with a time coupling constant of 1 ps. Simulations were carried out on an NVIDIA GTX980Ti processor and averaged about 50 ns/day for WT-Pin1. MD trajectories were analyzed using CPPTRAJ (39).

### Pearson correlation coefficients

Pearson correlation coefficients (*r* values) (60, 61) for pairwise parameters signifying intradomain and interdomain interactions were calculated as follows.

(Eq. 8) $$r=\frac{\sum_{i}^{N}\left(x_{i}-\langle x\rangle \right)\left(y_{i}-\langle y\rangle \right)}{\sqrt{\sum_{i}^{N}{\left(x_{i}-\langle x\rangle \right)}^{2}}\sqrt{\sum_{i}^{N}{\left(y_{i}-\langle y\rangle \right)}^{2}}}$$

The *r* values vary from −1 to 1, with 0 indicating no correlation. The variables *x* and *y* indicate distances or contact numbers: *x_i_* and *y_i_* are the individual snapshot values, 〈*x*〉 and 〈*y*〉 are averages over the entire trajectory, and *N* is the total snapshot count (*N* = 22,400). The estimated S.E. of *r*, denoted as S.E.*_r_*, is as follows,

(Eq. 9) $$\text{S.E}._{r}=\frac{\sqrt{1-r^{2}}}{\sqrt{N-2}}$$

where *r* is the correlation coefficient from Eq 8. For small *r* values, S.E.*_r_* is ∼0.007.

To further assess the significance of correlation coefficient *r*, we calculated the probability that *N* independent measurements of two uncorrelated variables would give an |*r*| ≥ |*r*_0_| (35, 36).

(Eq. 10) $$P_{N}\left(|r|\geq \left|r_{0}\right|\right)=\frac{2\Gamma \left[\frac{N-1}{2}\right]}{\sqrt{\pi }\Gamma \left[\frac{N-2}{2}\right]}\int_{\left|r_{0}\right|}^{1}dr{\left(1-r^{2}\right)}^{\frac{N-4}{2}}$$

Setting *n* = 22,400, we can find *P_N_* for various |*r*_0_| thresholds expressed as multiples of S.E.*_r_* above (Table 3).

**Table 3:** **P_N_ for various |r_0_| thresholds expressed as multiples of S.E.*_r_* (see**Equation 3)

*r*_0_	*P_N_*(|*r*| ≥ |*r*_0_|)
	%
1× S.E.*_r_* (=0.007)	29.5
2× S.E.*_r_* (=0.014)	3.6
3× S.E.*_r_* (=0.021)	0.2
4× S.E.*_r_* (=0.028)	0.0

Thus, to the extent that the *n* = 22,400 snapshots separated by 0.2 ns are independent samples, the probability that two uncorrelated variables would yield, by chance, *r* values ≥4·S.E.*_r_* (0.028) is highly unlikely. In other words, a correlation coefficient magnitude (absolute value) ≥ 4·S.E.*_r_* (0.028) is significant. We were more conservative, considering as significant only those correlation coefficients with magnitudes ≥0.05 (∼7·S.E.*_r_*).

### Hydrogen bond analysis

We defined the average occupancy of an H-bond between a pair of residues *X* and *Y* by its average occurrence over the entire trajectory (or the fraction of frames the H-bond is present). The distance and angle cutoffs for H-bonds were 3.2 Å and 135˚. Our metric was the sum defined as follows,

(Eq. 11) $$\Pi_{XY}=\sum_{i}^{N}O_{i,XY}$$

where *O_i_*_,_*_XY_* is the specific average occupancy of the *i*th H-bond over the trajectory, and *N* is the total number of H-bonds between residues *X* and *Y* (38) (Fig. 11).

## Data availability

Data are available upon request. Please contact Jeffrey W. Peng (jpeng@nd.edu) at the University of Notre Dame.
