# Supplementary material for: Coupled intra- and interdomain dynamics support domain cross-talk in Pin1
Source: J Biol Chem. 2021 Jan 13;295(49):16585–603. doi: 10.1074/jbc.RA120.015849 (PMC7864058; doi:10.1074/jbc.RA120.015849)
Supplement: Supplementary file 1 [file mmc1.pdf]

Coupling of inter- and intradomain conformation in Pin1 Investigated by Paramagnetic Relaxation Enhancements and Molecular Dynamics Simulations

Meiling Zhang[1], Thomas E. Frederick[1], Jamie VanPelt[1], David A. Case[2], Jeffrey W. Peng[1]*

From the [1]Department of Chemistry and Biochemistry, University of Notre Dame, 251 Nieuwland Science Hall, Notre Dame, IN 46556, USA; [2]Department of Chemistry and Chemical Biology, Rutgers University, 174 Frelinghuysen Road, Piscataway, NJ 08854, USA

CONTENTS (7 pages):

Table S1:	Summary of Residues Responses Upon Addition of MTSL Labels	(pg. 2)
Figure S1:	Similarity of Backbone ^{15}N Relaxation rates for WT and 3m-Pin1	(pg. 3)
Figure S2:	PREs predicted from Pin1 crystal structure 1PIN	(pg. 4)
Figure S3:	Histograms for PRE-related Interdomain Distances Sampled by MD	(pg. 5)
Figure S4:	Correlation coefficient histograms for interdomain distances and contact numbers	(pg. 6)
Figure S5:	Differential contact map for the two-cluster model	(pg. 7)

Table S1. Residue responses upon addition of paramagnetic MTSL and diamagnetic Acetyl-MTSL labels.

Location	Residues disappearing upon addition of para-MTSL	CSPs upon addition of dia-Acetyl-MTSL
WW Domain	G10	Y
	W11	Y
	E12	Y
	K13	Y
	F25	Y
	N26	Y
	T29	Y
	N30	Y
	A31	Y
PPIase Domain	S98	N
	F103	N
	G148	N
	F151	N

Note: Y and N denote the presence and absence, respectively, of ^{15}N-^{1}H CSPs upon addition of the label.

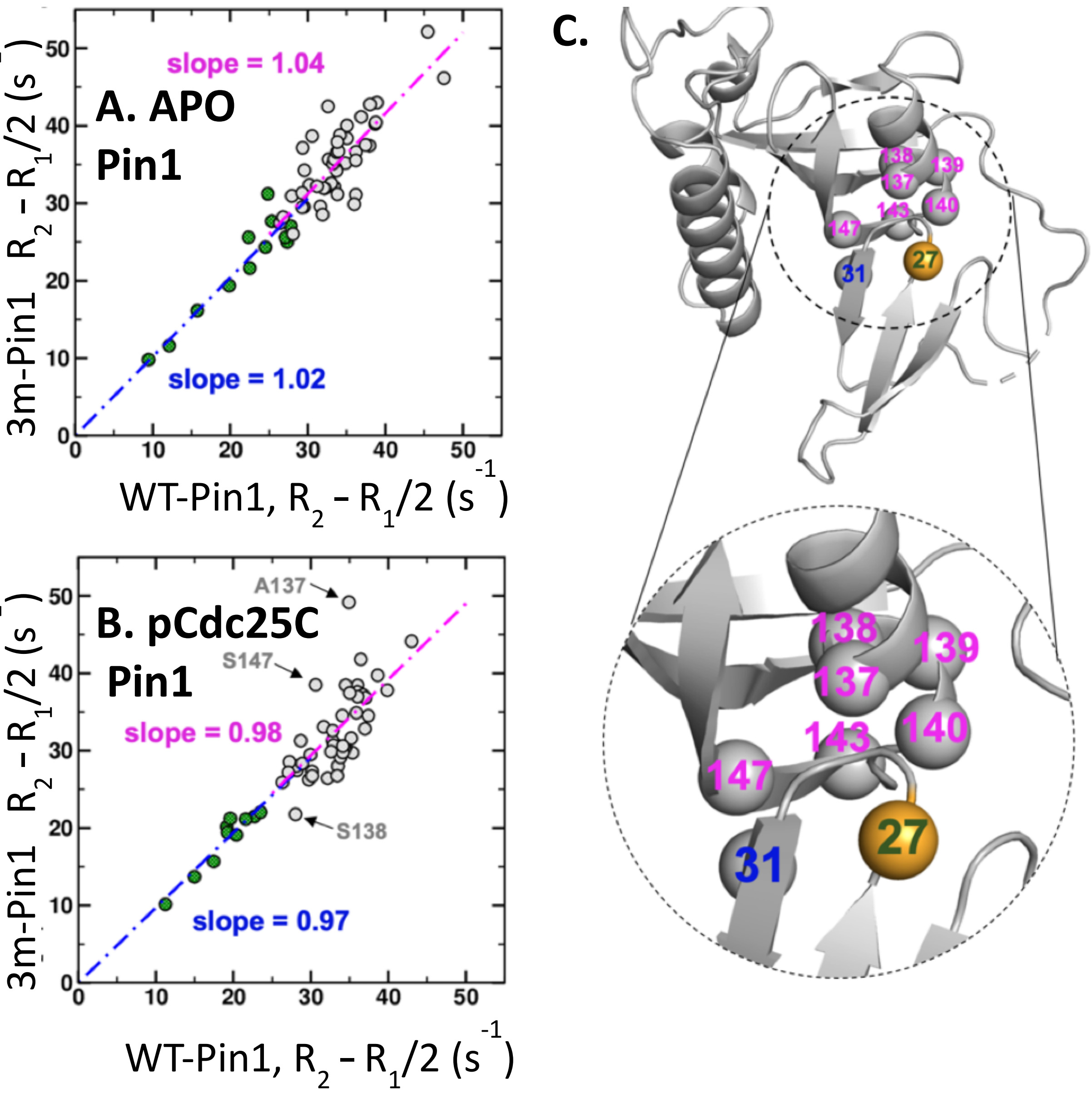

Figure S1. 3m-Pin1 preserves WT-Pin1 behavior. Linear correlation of backbone $^{15}N^{H}$ R_2-$R_1/2$ for WT-Pin1 versus 3m-Pin1 in the apo and pCdc25C-complexed states. Green dots denote WW domain residues, grey dots are PPIase domain residues. **(A)** apo WT-Pin1 versus apo-DIA 3m-Pin1, WW domain: slope = 1.02, correlation coefficient = 0.99; PPIase domain: slope = 1.04, correlation coefficient = 0.99 **(B)** Cdc25C-complexed WT-Pin1 ([pCdc25C]:[protein] = 12:1) versus Cdc25C complexed-3m-Pin1 ([pCdc25C]:[protein] > 12:1) WW domain: slope = 0.97, correlation coefficient = 1.00; PPIase domain: slope = 0.98, correlation coefficient = 0.99. (**C**) Residues with R_2-$R_1/2$ deviating significantly from the linear fit localize to the interdomain interface in the 1PIN crystal structure.

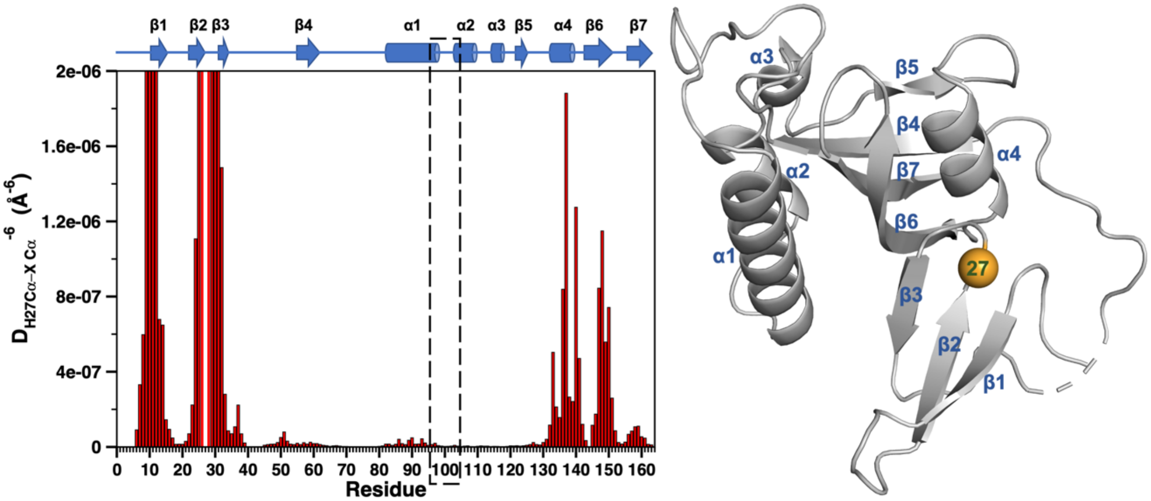

Figure S2. PRE profile of apo Pin1 predicted from the crystal structure 1PIN. Left: bar graph of per residue $D_{H27C\alpha\text{-}XC\alpha}{}^{-6}$, where $D_{H27C\alpha\text{-}XC\alpha}$ is the distance between the Cα of H27 and residue X extracted from the crystal structure of apo Pin1 (**PDB: 1PIN**). Note that WW domain residues P9-E12, F25-N26, and I28-A31 have $D_{H27C\alpha\text{-}XC\alpha}{}^{-6} > 2*10^{-6}$ Å^{-6} and thus are depicted as "over-flow" bars. $D_{H27C\alpha\text{-}XC\alpha}{}^{-6}$ is a good estimate of the $\Gamma_2(^1H^N)$ of residue X (See **EXPERIMENTAL PROCEDURES**). The black dash box shows the region that have small $D_{H27C\alpha\text{-}XC\alpha}{}^{-6}$ yet largest experimental $\Gamma_2(^1H^N)$ (see **Fig. 4**) highlighting the limitation of the crystal structure. **Right**: crystal structure of apo Pin1 (**PDB: 1PIN**). Residue H27 is denoted as an orange sphere.

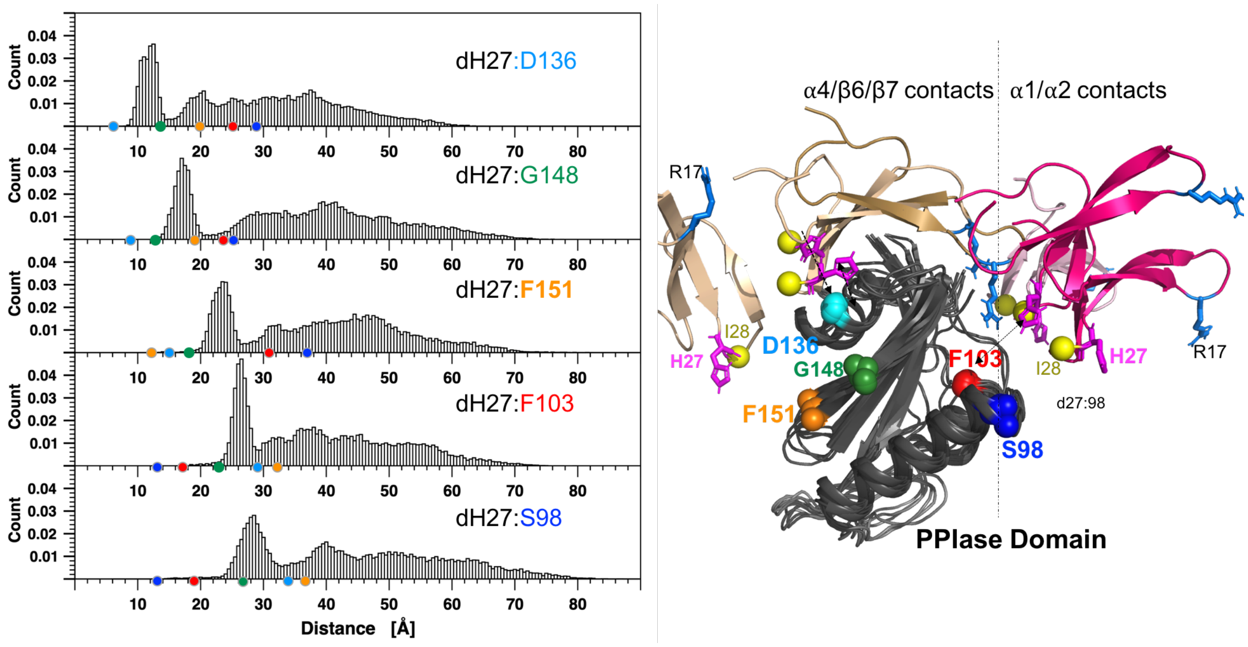

Figure S3. MD simulation of Apo WT-Pin1 captures interdomain conformations supporting the PREs of apo Pin1. (**Left**) Histograms of interdomain distances between H27 (MTSL attachment site) and the five PPIase residues (S98, F103, D136, G148, and F151) showing the most prominent PREs in the apo PARA 3m-Pin1 sample. (**Right**) Apo Pin1 MD configurations aligned by their PPIase domain (dark grey). The configurations correspond to the closest approach by the Cα of the five PPIase residues in the histograms to H27Ca. The configurations are distinguished by WW domain colors: (wheat) G148Cα and F151Cα; (hot pink) S98Cα and F103 Cα; (sand) D136 Cα.

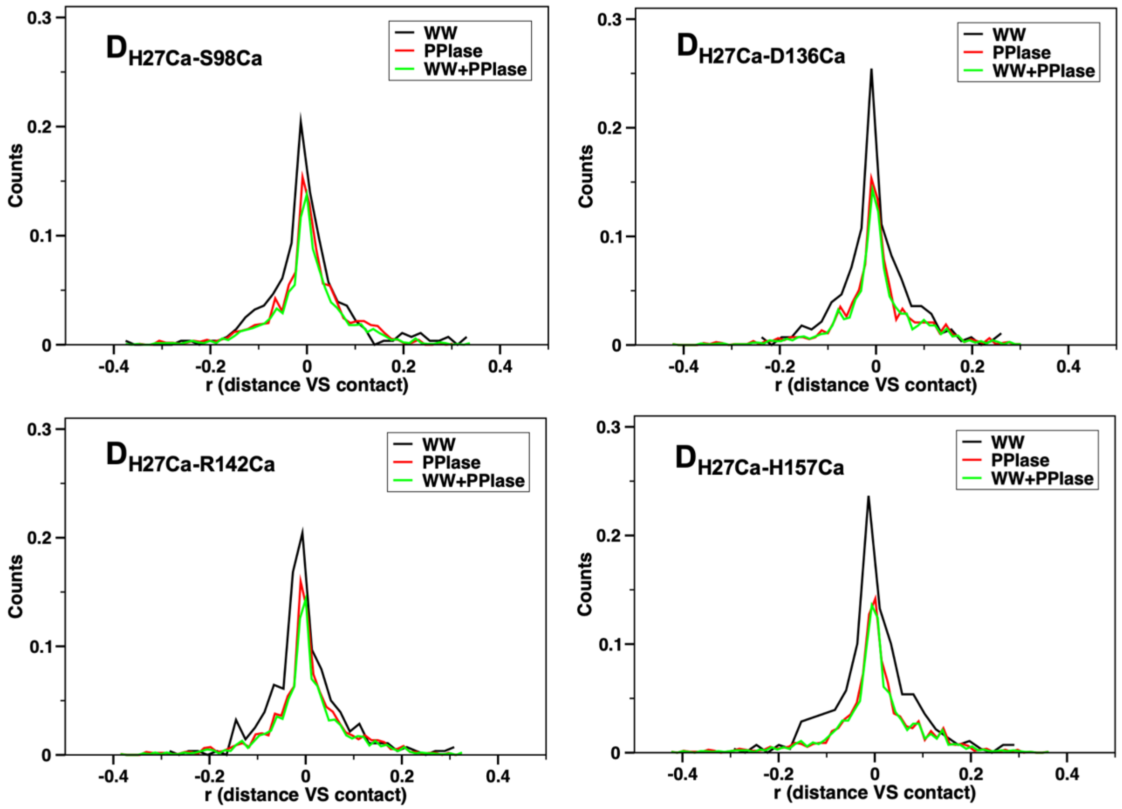

Figure S4. Correlation coefficient histograms for interdomain distances and contact numbers. Histogram of Pearson's correlation coefficient of the four interdomain distances with the intradomain contact within the WW domain (black), the PPIase domain (red), and the full-length Pin1 (green). The histograms are approximately symmetric about 0 (no correlation).

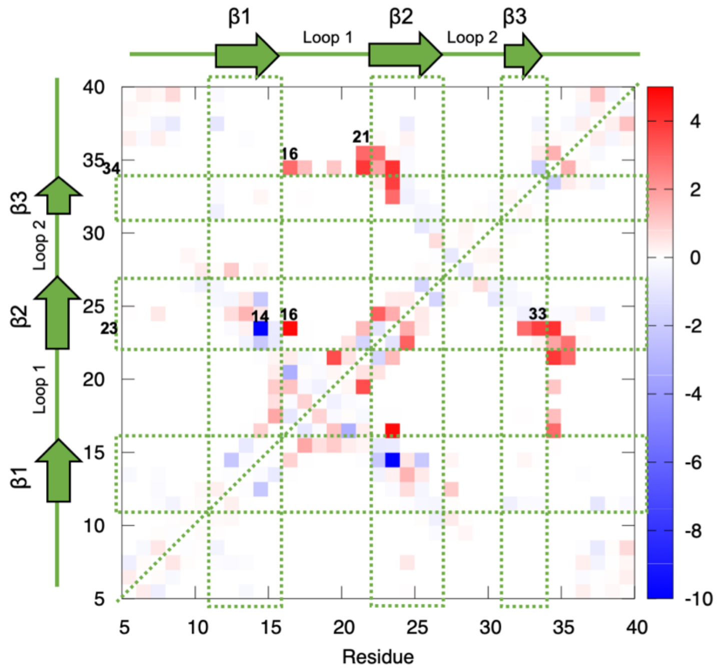

Figure S5. Differential contact map. Differential contact numbers between the two clusters representing the extended and compact forms of Pin1 ($Cluster_{Extended}$-$Cluster_{Compact}$). Positive (red) and negative (blue) indicated gain and loss of inter-residue contacts in the extended form of Pin1 relative to the compact form.
